# Swedish Well-Being: The rising importance of age among demographic, personality, and social relationship factors

**DOI:** 10.1016/j.ssmph.2026.101913

**Published:** 2026-04-01

**Authors:** August Håkan Nilsson, Petri J. Kajonius, Oscar Kjell, Micael Dahlen, H. Andrew Schwartz, Brendan Case, Byron Johnson, Tim Lomas, Noah Padgett, Tyler J. VanderWeele

**Affiliations:** a Oslo Business School, Oslo Metropolitan University, Oslo, Norway; b Department of Psychology, Lund University, Sweden; c Department of Clinical Research, Lund University, Sweden; d Center for Wellbeing, Welfare and Happiness, Stockholm School of Economics, Sweden; e Department of Computer Science, Stony Brook University, USA; f Human Flourishing Program, Institute for Quantitative Social Science, Harvard University, Cambridge, MA, USA; g Department of Epidemiology, Harvard T.H. Chan School of Public Health, Boston, MA, USA; h Institute for Studies of Religion, Baylor University, TX, USA; i School of Public Health, Harvard University, Cambridge, MA, USA

## Abstract

The main demographic and psychological correlates of Well-Being (WB) are well-established, but have not yet been assessed in the Swedish population–regularly ranked among the world's top five ranked WB nations–based on large, nationally representative data. Using 2023 Global Flourishing Study data (*N* = 15,068), this paper analyzes Swedish WB across three domains: demography (e.g., gender, age, and income), individual personality traits (e.g., Big Five neuroticism and extraversion), and social relationship qualities (e.g., loneliness and relationship satisfaction). Using machine learning regression models based on these three domains, we regressed a composite 13-item WB measure–i.e., a general factor consisting of positive markers (e.g., happiness, life balance) and negative markers (e.g., depression, anxiety)--with an accuracy of *r* = .79 (cross-validated training) that generalized well to a holdout set (*r* = .79). Age was the strongest demographic marker of Swedish WB among the 65 predictors (lasso *β* = .10; bivariate *r* = .32), only surpassed by the classically strong WB predictors of neuroticism (*β* = −.33), loneliness (*β* = −.24), relationship satisfaction, (*β* = .17), and friendship contentment (*β* = .17). We replicated the age–WB relationship with complementary Gallup World Poll data; spanning 2006 to 2024, and the data suggested that older Swedes have indeed pulled ahead of young Swedes in WB, albeit only in the last five years. Further, with this paper's focus on the demographics of WB in Sweden, it offers an unprecedented set of political identity graphs for the benefit of researchers, policymakers, and the common public. The overall conclusion is that while personality and social relationship quality are stronger markers than demography for Swedish WB, age is the strongest demographic predictor, and has grown in significance recently. The findings will ideally inform and guide Swedish public policy and politics, particularly in addressing the declining WB of young Swedes.

## Highlights

• We assessed Swedish Well-Being (WB) from two national datasets (*N* = 30 K+).
• Models combining demography, personality, and relationships predict WB (*r* = .79).
• Social relationships and neuroticism were the strongest predictors of Swedish WB.
• Age has gone from a zero to medium-strong WB predictor in the past few years.

## 1 Introduction

Sweden consistently ranks among the highest-scoring nations for well-being (WB), such as in the latest World Happiness Report (Helliwell et al., 2025), where Sweden placed number four, and is a world outlier in the World Value Survey cultural map (World Value Survey, 2023). While WB is becoming a governmental priority alongside and perhaps even instead of GDP (Mahoney, 2023) and official Swedish statistical agencies have started to collect yearly WB reports in Sweden (Statistics Sweden, 2023a, 2024a, 2025a), these official metrics often rely on single-item WB metrics. Consequently, there remains a scarcity of large-scale, representative research focusing on multidimensional WB in Sweden and its deeper psychological markers, especially beyond basic demographics (with partial exceptions such as Fors Connolly & Gärling, 2024; Hansson et al., 2005; Winzer et al., 2021). Considering that Sweden is among the happiest and culturally most extreme countries in the world (World Value Survey, 2023), with one of the world's largest and long-standing welfare states (with, for example, free health care and education; 1177 healthcare guide, 2023; Eurydice, 2023), and enjoys one of the highest levels of social trust globally (Delhey & Newton, 2005), the country offers a unique and special case for studying WB and its markers. The present study used Wave 1 Global Flourishing Study (GFS) data, together with Swedish Panel Data (*N* = 15,068), to analyze predictors of WB in Sweden based on three well-established factor domains: demographic characteristics (e.g., gender, age, and socioeconomics), individual characteristics (e.g., Big Five personality traits), and social relationship characteristics (e.g., loneliness and relationship satisfaction). To our knowledge, this is the first nationally representative study focusing exclusively on Swedish WB in relation to demographics while controlling for psychological and social correlates.

### 1.1 Well-being

In psychological research, the most common lens through which WB, and related concepts like happiness, are operationalized is “Subjective WB” (SWB; Deci & Ryan, 2008). A classical definition of SWB is “the level of well-being people experience according to their subjective evaluations of their lives” (*p*. 19, Diener & Ryan, 2009). SWB is typically seen as composed of a cognitive and an affective component (Diener, 1984). The cognitive component refers to an individual's subjective evaluation of their life, and is commonly conceptualized and assessed through either life satisfaction (such as the Satisfaction with Life scale; Diener et al., 1985) or life evaluation (through the Cantril Ladder, used in the World Happiness Report, Cantril, 1965). The affective component consists of positive and negative affect (Watson et al., 1988), including how individuals experience emotions such as happiness, stress, and anxiety. However, this standard two-component model has been criticized for only capturing and measuring a narrow, hedonic, and Western-centric view of SWB (Ryan & Deci, 2001), and recent research has broadened its operationalization by including, for example, Harmony in Life, Peace and Balance (Kjell et al., 2016; Lee et al., 2013; Lomas et al., 2022). Then, in addition to SWB, somewhat different WB-related territory is captured by the idea of eudaimonic WB (EWB), involving components such as purpose and meaning, as emphasized by philosophers such as Aristotle, who argued that eudaimonia was the end goal of human existence (Aristotle, 1985). However, although some scholars sometimes argue that EWB is distinct from SWB (Martela & Sheldon, 2019), others argue that the distinction should instead be between eudaimonic and *hedonic* WB (Keyes, 2006). Additionally, there are broader concepts such as mental health–to which increasing attention has been paid in recent years, especially when considering how young people are faring (Twenge et al., 2022)–which might be seen as encompassing different WB types. Thus Keyes, for example, defines mental health as “a complete state of subjective well-being (i.e., hedonic and eudaimonic well-being) as well as the absence of common mental disorders” (*p*. 7, Keyes, 2006). In this study, we allow for a broad definition of WB, encompassing hedonic and eudaimonic WB, positive and negative affect, and mental health.

### 1.2 Demography and well-being

The first WB domain, and the particular focus of the present study, is demographic characteristics. In one of the more exhaustive longitudinal meta-analyses, based on 443 studies following almost half a million participants across their lives, life satisfaction (the cognitive component of Subjective WB) was relatively stable over the lifespan (although it decreased during early teenage years), while positive affect decreased and a u-curve^1^ was observed for negative affect (with increased negative affect after 60; Buecker et al., 2023). Looking at cross-sectional studies, findings have also suggested a U-curved relationship between age and life satisfaction, where life satisfaction dips in midlife (Blanchflower, 2020), although this trend has turned linear in many countries in recent years (Blanchflower et al., 2025). Thus, longitudinal and cross-sectional data show different patterns, where some of the U-shaped curves observed in cross-sectional data might relate to cohort effects. Although findings are generally mixed across study types in meta-analyses across countries, the age-WB relationship has been close to 0 both cross-sectionally and longitudinally between 1982 and 2009 in Sweden for evaluative WB (Realo & Dobewall, 2011). However, recent data from Swedish Statistics suggest a positive relationship between age and evaluative WB up until 65-74 years, after which WB decreases again (Statistics Sweden, 2023b). The 2024 World Happiness Report devoted a full chapter to WB and age, in which they showed that people aged 30 and below had the lowest evaluative WB–based on a measure of life evaluation, the Cantril Ladder–in only 7 out of 143 countries, of which one was Sweden (Helliwell et al., 2024). Thus, there is reason to expect that the current study data will show a meaningful relationship between age and WB.

Turning to other demographic characteristics, in Western societies, women report slightly higher evaluative WB than men on the Cantril Ladder across various ages cross-sectionally (Steptoe et al., 2015). Socioeconomic status (SES) or social class is also among the most general demographic and societally relevant markers of population WB. One prominent meta-analysis (Tan et al., 2020; k = 357) reached the following conclusions: subjective (psychological) SES is more related to WB than objectively measured SES, although this discrepancy is partly explained by common method variance (i.e., SES and WB are both assessed by self-reported rating scales, unlike income, which, if self-reported, is reported in currency levels rather than a typical likert scale). Furthermore, income and wealth tend to drive the relationship with SWB more than education. The SES-WB link also seems to diminish with income equality and increase with mean national wealth level for happiness and evaluative WB (Tan et al., 2020), suggesting that the association might be weaker in Sweden given its relatively high levels of income and income equality. Several large scale studies suggest a linear, robust but weak relationship between income and evaluative and emotional WB (Killingsworth, 2021; Killingsworth et al., 2023). In general, socioeconomics are expected to predict WB, but less than individual characteristics or relationships with friends, as reported in a previous meta-analysis (Pinquart & Sörensen, 2000).

Turning to politics, to date, we are not aware of any research that has mapped WB to Swedish political party identities. Extensive research elsewhere–particularly the USA–has focused on the liberal-to-conservative continuum in relation to WB, where conservatives have shown higher WB (Napier & Jost, 2008; Okulicz-Kozaryn et al., 2014; Stavrova & Luhmann, 2016). However, the one dimensional liberal–conservative continuum is American-centric and not well suited for Sweden, a very socialist country, where *economic left to right* might better be separated from *cultural liberalism (*or *Green, Alternative and Liberal; GAL) vs conservatism (or Traditional, Authoritarian, and Nationalistic; TAN;* Wheatley & Mendez, 2021*)*. Sweden has eight parties in the parliament, with the classically economic far left being culturally liberal and high on GAL (e.g., the Left Party and the Green Party) and the classically middle left, with the biggest party (The Social Democrats), being more towards the middle on both cultural liberalism (i.e., GAL) and governmental influence on the economy. The classically middle right is more towards cultural liberalism (i.e., GAL) and free market (the Centre Party and the Liberals), with the classically right parties being more culturally conservative (TAN) and promoting free market ideals (the Moderates and Christian Democrats). The classically far right (the Swedish Democrats) is culturally conservative (high on TAN) but middle in governmental influence on the economy (Björkman, 2020; Bäckersten, 2022; Rovny et al., 2025). In sum, several demographic domains might explain Swedish SWB. However, we will evaluate the demographics next to two of the strongest SWB predictor domains, personality and social relationships.

### 1.3 Personality and well-being

One of the age-old questions in WB research is whether demographic life circumstances such as gender, age, and socioeconomics lead to WB, or whether WB is mostly already inherent in terms of personality traits and perceived quality in one's social interactions. Longitudinal survey studies and experimental evidence in meta-analysis lean towards favouring personality and social interactions (Lyubomirsky et al., 2005), implying that WB is ultimately rooted in what could be called “happy personality” (Busseri & Erb, 2024). From the cross-sectional perspective, using meta-analytic structural modeling, the temperament traits of neuroticism, extraversion, and conscientiousness in particular have been shown to be strong correlates of general WB in life (Anglim et al., 2020). In Sweden, neuroticism has been a particularly strong marker for low emotional WB (Fors Connolly & Gärling, 2024) and general WB in adolescents (Winzer et al., 2021). However, the personality–WB relationship seems to be mainly explained by common genetics, with reports of almost no unique genetic WB variance left to account for in the presence of personality, i.e., the ubiquitous Big Five model (Weiss et al., 2008). Usually, heritability for WB is in the 30-40% range; however, separating the stable lifetime variance across measurement types in longitudinal models yields heritability in the 60-70% range (Nes & Røysamb, 2017). This indicates that general WB is somewhat stable throughout life and that changes or fluctuations from the individual's baseline are mostly situationally driven. It should be noted, however, that parts of the personality–WB may be attributed to common method variance (Podsakoff et al., 2024). For example, social desirability has shown to explain around half of the personality–WB relationship (Persson et al., 2025).

### 1.4 Social relationships and well-being

Another domain of strong WB predictors is social relationships, which at least partly is separated from one's personal characteristics. Using large experience sampling methods, the data has shown that social relationships are amongst the most regular and effective ways people use to modify their moods (e.g., to help raise it after a fall; Quoidbach et al., 2019). When evaluating pre-registered studies on popular WB interventions, being more sociable is one of the few interventions that seem to boost at least emotional WB (Folk & Dunn, 2024). Among various types of social relationships, romance and marital relationships in one longitudinal meta-analysis (k = 78) were most related to WB in the social domain (Proulx et al., 2007). Further complicating the picture, however, is the dramatic shift over the past decade towards moving social interactions online, and research is still accumulating data to understand how these digital versions of social qualities may affect WB broadly. One recent meta-analysis concluding mostly zero-associations in general WB measures (Marciano et al., 2024), although the strongest (negative) association was social media use as social comparison, with social comparisons being an established negative predictor of WB (Luttmer, 2005)–a finding for which evidence continues to mount (Capraro et al., 2025).

### 1.5 Previous studies on well-being in Sweden

While previous studies have established foundational predictors of Swedish WB, comprehensive research combining large, representative samples with deeper psychological markers is rare. An example is a study from 2024 focusing on the differences in predicting life satisfaction and emotional WB, where neuroticism was shown to be a particularly strong predictor of emotional WB (Fors Connolly & Gärling, 2024). The most similar study to the present is one from 2004, which assessed a large representative sample limited to Stockholm (the Swedish capital), and could explain 20% of the WB variance in Stockholm, with friendship support being the strongest predictor, followed by the absence of negative life events and financial problems, and with age having a small positive association with WB (Hansson et al., 2005). In another longitudinal study focusing partly on Sweden, there was no age–WB relationship cross-sectionally or longitudinally between 1982 and 2009 in Sweden using nationally representative data (Realo & Dobewall, 2011). More recently, a contemporary descriptive paper utilizing the same broader dataset as the present study mapped baseline demographic disparities in Swedish flourishing and showed a strong age association (Bittár et al., 2025).

## 2 Present study

The present study set out to understand what constitutes Swedish WB. We made use of GFS data (available at the Center for Open Science) for cross-sectional analysis, together with Gallup World Poll (GWP) data for longitudinal analysis, and focused on predicting a broad composite measure of WB based on three domains, both separately and combined: demographic characteristics (e.g., gender, age, and socioeconomics), individual personality characteristics (e.g., Big Five neuroticism and extraversion), and social relationship characteristics (e.g., loneliness and relationship satisfaction). This aim of quantifying explained SWB among Swedish people is arguably the first reported study using representative panel data. We aim to evaluate.

1. How demographic characteristics relate to WB in Sweden.
2. The important predictors of WB in Sweden among demographics, personality and social relationships.

## 3 Method

The description of the methods below regarding the GFS has been adapted from VanderWeele et al. (2025). Further methodological detail is available elsewhere, including an overview of the GFS as a whole (Johnson et al., 2024) and its general methodology (Ritter et al., 2024); an initial questionnaire development report (Crabtree et al., 2021), as well as an updated account of the questionnaire development process, (Lomas, Bradshaw, et al., 2024), of which one aspect was a process piloting the items through cognitive interviewing, (Cowden et al., 2024); the Wave 1 codebook (Markham et al., 2024); the survey sampling design for Wave 1 (Padgett, Cowden, et al., 2024); the initial statistical analyses code (Padgett et al., 2024a, Padgett et al., 2024b, Padgett et al., 2024c); the analytic methodology for demographic variation analyses for wave 1 (Padgett, Bradshaw, Chen, Cowden, et al., 2024a). The current paper, which focuses specifically on Sweden, was pre-registered on May 12th, 2025 (https://osf.io/f84cv/overview). The data are publicly available through the Center for Open Science (https://www.cos.io/gfs).

### 3.1 Data

The GFS is a study involving – in its first wave in 2023 – 207,919 participants from 22 geographically and culturally diverse countries, including Sweden, with nationally representative sampling within each country, and is concerned with the distribution of determinants of WB. Data collection was carried out by Gallup. Data in Sweden for Wave 1 were collected in January (26%), February (43%), and March (30%) of 2023 (Ritter et al., 2024). The precise sampling design to ensure nationally representative samples varied by country, and further details are available (Ritter et al., 2024). For Sweden, non-probabilistic, pre-existing, digital opt-in panels were recruited by Gallup. Sweden is one of the most digitalized countries in the world (IMD World Competitiveness Center, 2024), and thus, there should not have been a major self-selection bias arising from surveys being digital. Then, pseudo-sampling weights based on propensity score methods were calculated for Sweden using official statistics for the variables age ∗ gender, education and region (for details, see Ritter et al., 2024). The approach estimated the probability of inclusion in the panel frame based on the demographics and generated respondent-level survey weights for subsequent analysis. Sample characteristics are shown in Table 1 for age and gender compared to the Swedish population. The average adult age in Sweden was 49.9 years^2^ in 2023 (Statistics, 2024b), and the weighted average in the study data was 49.4 years. The gender distribution in Sweden in 2023 was 49.7% women (Statistics, 2025b), and the weighted distribution in the study data was 49.4%. Thus, the study data's basic demographic composition mimicked official Swedish statistics to a large degree, already without the weights, but even more with the weights. Still, as the sample was collected through non-probability based opt-in panels, we highlight that the data is, with the weights applied, *proportionally* representative, as we can not rule out the possibility of self-selection bias for groups such as older Swedes with low digital literacy.

**Table 1:** Sample characteristics of Swedish adults, officially and in the study sample.

	Mean Age	% women
Official statistics	**49.9 (19.3)**	**49.7%**
*Total sample*		
Weighted	49.4 (18.7)	49.9%
Non-weighted	48.5 (19.0)	49.0%
*Training*		
Weighted	49.4 (18.7)	49.5%
Non-weighted	48.5 (19.1)	48.9%
*Holdout*		
Weighted	49.3 (18.5)	51.3%
Non-weighted	48.6 (18.8)	49.6%

*Note*: Standard deviation in brackets. After applying population weights, 0.2% of participants in the total sample identified with a gender other than male or female. The oldest person in the Swedish sample was 98 years old.

### 3.2 Measures

#### 3.2.1 Criteria

**Fig. 1:**
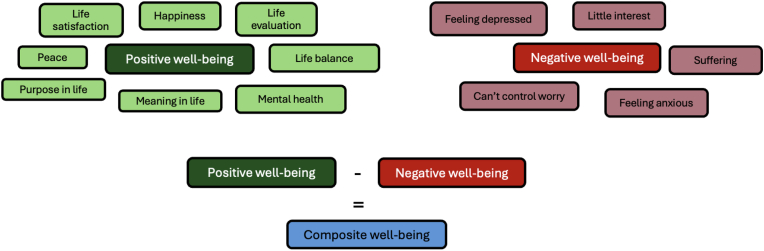
Composite well-being.

The items this paper focused on revolved around WB as the outcome, with demographics, personality traits and social relationship quality as predictor variables. The WB measures were grouped into eight positive WB indicators and five negative WB indicators (Fig. 1). The positive WB variables were happiness, life satisfaction, life evaluation, peace, balance in life, meaning, purpose and mental health. The negative WB indicators were two depression indicators (Feelings of depression and Having little interest), two anxiety indicators (feeling anxious and not being able to control worry), and suffering (for a complete list of the items, see Supplemental Material Table S1).

We computed a positive and negative WB composite by averaging the standardised scores of these eight and five measures, respectively. The items had a Cronbach's Alpha of .93 and .88 and McDonald's Omega of .96 and .91 for positive and negative WB, respectively. The average intercorrelation between the eight positive WB variables was *r* = .62 (*SD* = .11) and *r* = .59 (SD = .11) for the five negative wb variables.

By themselves, the positive and negative WB composites were multidimensionally capturing WB. We took one step further and subtracted the negative WB from the positive WB to encompass both sides of WB and standardised the final composite WB (see Fig. 1). It is typical to include multiple aspects of WB to capture more aspects of WB (e.g., Romero et al., 2012). By using a multidimensional assessment of WB, the outcome captured hedonic, eudaimonic, affective, and mental health components of WB. The Cronbach's alpha for these 13 WB variables was .94, and the McDonald's Omega was .96. The average absolute intercorrelation between these variables was *r* = .55 (*SD* = .11). A heat map of the intercorrelation between the WB indicators and the composite scores can be found in the Supplemental Material Fig. S13. The resulting composite WB score was used as the main outcome of the study paper.

#### 3.2.2 Predictors

**Fig. 2:**
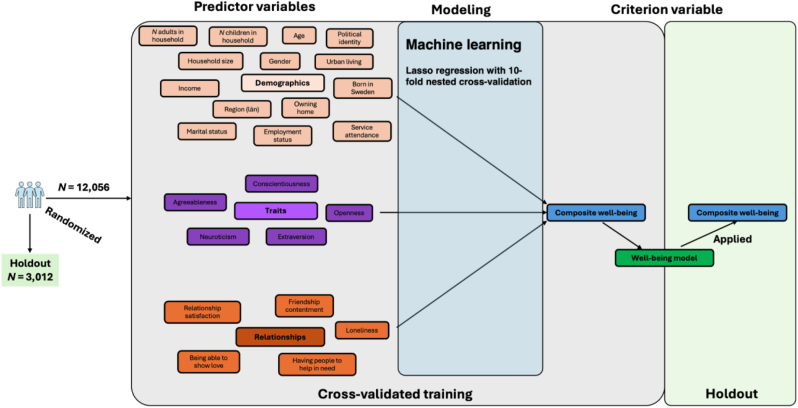
Analysis pipeline of Well-Being models.

Next, we present the predictor variables, which were grouped into demographics, personality traits, and social relationship quality (see Predictor variables in Fig. 2). Regarding demographics, age was reported in years. For gender, we made it binary, as fewer than .3% had indicated “other”. Income was reported in 12 categories starting from “less than 10,000 SEK”, increasing by a factor of 5000 SEK up to 40,000 (except for a 10,000 increase from 15,000 to 25,000), then by a factor of 10,000 SEK up to 80,000, then up to 100,000 SEK and the last category was above 100,000 SEK. The region was in terms of the 21 Swedish counties (rather than the 24 provinces). Marital status was assessed as single/never married, married, separated, divorced, widowed, and domestic partner. Employment was assessed as employed, self-employed, retired, student, homemaker, unemployed and searching, and other. Education was assessed in eight levels where we grouped “no formal education”, “Incomplete primary school”, and “Primary education” into one group. The remaining five steps followed International Standard Classification of Education (ISCED) level from 2 to 6 with “Tertiary education – advanced level” as the highest level. Religious service attendance was assessed as more than once/week, once/week, one-to-three times/month, a few times/year, or never. Immigration status was dichotomously assessed with: “Were you born in this country, or not?” Religious tradition/affiliation with categories of Christianity, Islam, no religion/atheist/agnostic and 12 other categories (e.g., Buddhism) being more than .0001% of the data, and we thus excluded them. For additional details on the assessments, see the COS GFS codebook (Markham et al., 2024) or Crabtree et al. (2021).

Personality traits were assessed by the Ten-Item Personality Inventory (TIPI; Gosling et al., 2003) with two items for each of extraversion, openness to experience, agreeableness, conscientiousness, and neuroticism, with one positive and one negatively phrased item. They were rated on a scale from 1 = Disagree strongly to 7 = Agree strongly.

Social relationship quality was assessed by five items that were all rated on a 0-10 scale. They included relationship satisfaction, friendship contentment, social support, loneliness, and having people to show love. For the items of the predictor variables, see Supplemental Material Table S1.

All items were translated by Gallup. During the translation process, Gallup adhered to the TRAPD model (translation, review, adjudication, pretesting, and documentation) for cross-cultural survey research; for additional details, see the questionnaire development process report (Lomas, Case, et al., 2024).

### 3.3 Analyses

#### 3.3.1 Well-being prediction models

Our first analysis involved assessing the composite WB score from the demographic information, personality traits, and social relationship quality of the participants (see Fig. 2). We applied the machine learning algorithm Lasso regression and used weighted nested cross-validation in the training of the Lasso regression (see Kjell et al., 2023). Although nested cross-validation is a robust approach to secure generalisability of machine learning models, an additional robustness test of generalisation is applying the model on a holdout set that was not part of the training. Thus, the first step of the cross-validation procedure involved splitting the data into 80% training and 20% holdout, which we did with reproducible randomness stratified for the outcome variable (the composite WB measure). The difference in age, gender, and marital status between training and holdout sets can be seen in Table 1. We refer to this training and holdout split as *first-level training and holdout*.

Next, we trained several Lasso regressions using 10-fold nested cross-validation using various amounts of predictor variables. A Lasso regression is conceptually similar to a standard multiple regression. However, whereas a normal multiple regression is sensitive to correlated predictors (multicollinearity), the Lasso is more robust. The Lasso regressions include a penalty that pushes predictor variables that are not useful for the model to 0. Given that our full model included 65 predictor variables, we found the Lasso strategy most useful for interpreting the model and its beta coefficients. The optimal penalty level is determined using a nested cross-validation procedure. The first step in cross-validation is splitting the data into 10 chunks. Each of the 10 chunks is once excluded to be the *second level holdout,* with the remaining 9 chunks being the *second level training*. In each of these 10 s-level splits, the 9 chunks are split up again into a *third-level training and holdout* that tests different penalties in its training, which is evaluated on the third-level holdout. The penalty with the best performance on this third-level holdout is then used with the 9 chunks representing the second-level training and applied on the second-level holdout. Since this procedure is repeated 10 times, all observations get an out-of-sample prediction, meaning they get a prediction from a model that never saw the data point.

The final model uses the penalty that performed the best across the 10 s-level holdouts (for more details about the nested cross-validation procedure, see Kjell et al., 2023). We used a penalty grid of 10^seq(1, −3)^, i.e., an element among 10, 1, .1, .01, and .001. All models got a penalty of .01 except for the model with relationship and personality, which had a penalty of .001. Having a penalty on the edge of the penalty grid might not reflect the optimal penalty. We thus tried to expand the grid for the personality + relationship model to include .0001 and .00001. The penalty remained at .001. Lastly, the out-of-sample predictions were correlated with the observed values using a standard Pearson correlation, and these numbers are presented in the results. What is also presented is the final models' performances on the first level holdout (also in terms of Pearson correlation), data that was never part of the training.

**Table 2:** Predicting Swedish Well-Being from demographics, social relationships and personality with machine learning.

Predictors	*N* predictors	Cross-Validated Training	Holdout
		Pearson *r* between predicted and actual well-being	
Demographics	55	.45 (*n* = 11,098)	.46 (*n* = 2,768)
Personality traits	5	.62 (*n* = 11,843)	.62 (*n* = 2,961)
Social relationships quality	5	.70 (*n* = 11,879)	.71 (*n* = 2,968)
Demographics + personality traits + social relationships quality	65	.79 (*n* = 10,779)	.79 (*n* = 2,688)

*Note:* Demographics include age, gender, region, political party orientation, income, employment status, number of adults in the household, number of children in the household, household size, marital status, urban vs rural living, born in Sweden/abroad, and religious service attendance. Personality traits include the big five traits. Social relationship quality includes friendship contentment, relationship satisfaction, loneliness, having people to help in trouble and having people to show love to. First, we looked at the participants' life circumstances (demographics), then added their internal traits (personality) and then added their social environments (relationships).

#### 3.3.2 Substantial well-being predictors

The Lasso gives us a nuanced view of how much the 65 predictors contributed in assessing WB in Sweden when all are considered concurrently. Since Lasso regression pushes redundant predictors down to 0, we show all non-zero beta coefficients from the final model. These beta coefficients reflect the strength of the specific predictor in the context of the other predictors and are all standardised (bivariate associations can be higher and are found in Supplemental Material Fig. S12).

**Fig. 3:**
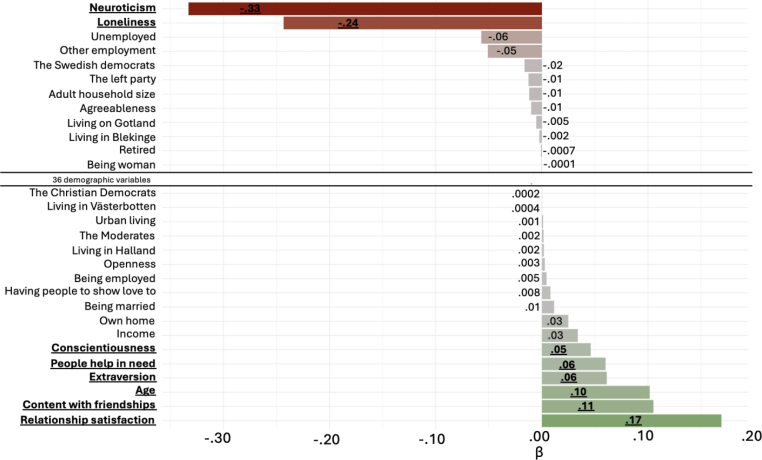
Beta (*β*) coefficients beyond 0 in the Lasso regression predicting Well-Being at *r* = .79 with penalty 0.01

We utilized classical thresholds of relationship effect sizes in psychology research (Cohen, 2013; Gignac & Szodorai, 2016) to comment principally on those with beta coefficients ≥.10 with .10 = small, .20 = medium, and .30 = strong based on Gignac and Szodorai (2016). Further, we indicate which predictors remained non-zero when we increased the Lasso penalty from .01 to .1 (which pushes close-to-zero beta coefficients all the way down to zero) by making them bold. The predictor variables and the WB criterion were all standardised before modelling.

#### 3.3.3 Highest and lowest predicted well-being

To further understand the model performance, we showcased the demographic and psychological characteristics of the three participants with the highest and lowest predicted WB in the first-level holdout set. They showcase examples of the extreme ends of high and low predicted WB in Sweden and were selected based on the predicted WB scores.

**Table 3:**
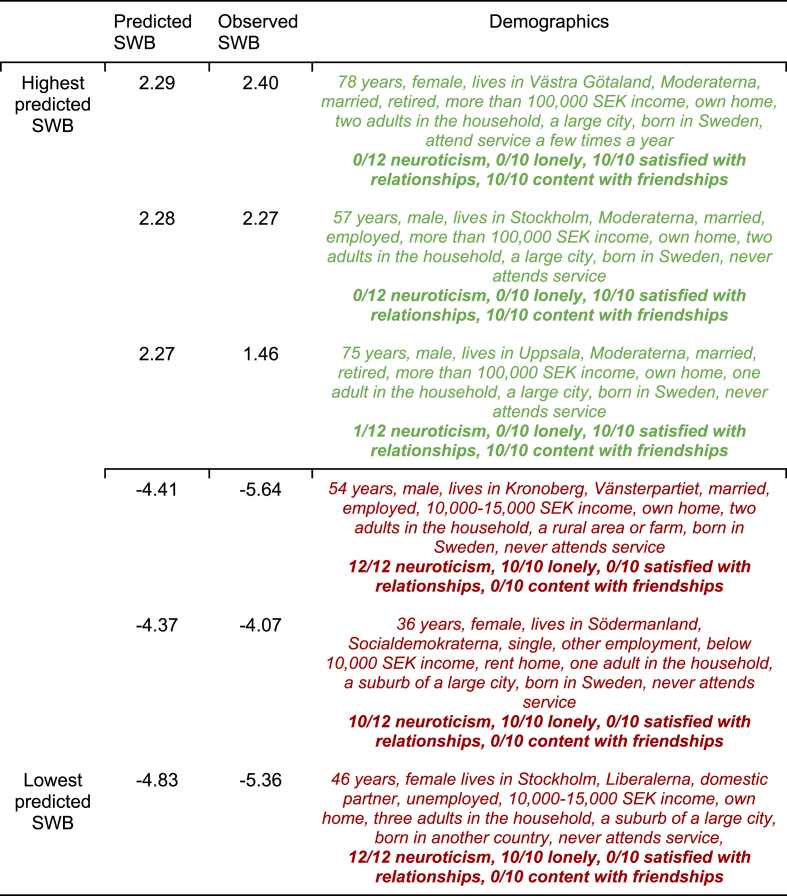
Demographics of the individuals with the highest and lowest predicted Well-Being.

*Note:* The participants in the holdout sample with the highest and lowest predicted WB from our most accurate machine learning Lasso regression (in Table 2) and their demographic characteristics and psychological scores on the strongest predictors. Bold descriptions are psychological variables.

#### 3.3.4 Well-being and age with the Global Flourishing Study data

We then moved beyond the prediction model and evaluated the full data set on the relationship between age and WB. We tested the relationship for the full sample and separate analyses for men and women, as well as the positive, negative, and composite WB (Fig. 2). We used weighted correlations in the full sample and Cohen's *d* between the five youngest years (18-23) and five oldest years (75-80). For the correlations, we used all the data as it was. For the figure and for Cohen's *d*, we grouped everyone above 80 into one age group since there were few participants for each age beyond 80.

#### 3.3.5 Well-being and age with the Gallup World Poll data

Based on the findings between age and WB in the GFS data, we wanted to replicate the findings and evaluate the age WB relationship in another dataset. We used the GWP (see Gallup, 2019) data that has collected one of the metrics in the composite WB metric (i.e., life evaluation) every year between 2006 and 2024 in a representative Swedish sample. Each year, around 1000 participants were included (range of 989–1013) except for 2013 (*n* = 750) and 2014 (*n* = 1990). For each year, we tested the weighted correlation between age and life evaluation and corrected for multiple comparisons with Benjamini-Hoshberg correction (Benjamini & Hochberg, 1995). We show the relationship between age and WB every year with a smoothed trend line, estimated using locally estimated scatterplot smoothing (LOESS).

**Fig. 4:**
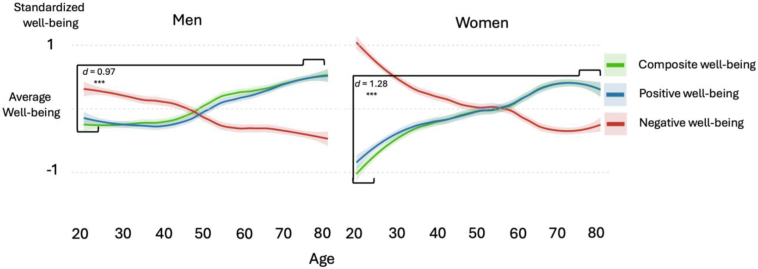
Well-Being across age and gender in Sweden

#### 3.3.6 Well-being and political party voting

Lastly, we evaluated the political party identity in relation to WB. We fitted a similar Lasso regression as in Table 1, only using dummy codings of political parties to estimate how much party orientation by itself related to WB. The final penalty was .0001. Then, we plotted the weighted WB means for each party, along with their average household income, average age and how many percentages identified with each party. We did similar figures for region, income and other demographic variables which can be found in the Supplemental Material.

##### 3.3.6.1 Inference

We interpreted effect sizes of correlations and beta coefficients at .10 = small, .20 = medium, and .30 = strong based on an empirical meta-analysis of effect sizes in individual differences research (Gignac & Szodorai, 2016).

##### 3.3.6.2 Accounting for complex sampling design

The GFS used different sampling schemes across countries based on availability of existing panels and recruitment needs (Ritter et al., 2024). All analyses accounted for the complex survey design components by including weights, primary sampling units, and strata. Additional methodological detail, including accounting for the complex sampling design is provided elsewhere (Padgett, Cowden, et al., 2024).

### 3.4 Data availability

Data for the GFS is available through the Center for Open Science after submission of a preregistration (https://doi.org/10.17605/OSF.IO/3JTZ8), and the data will be openly available without preregistration in 2026. For additional information about data access see the COS website (https://www.cos.io/gfs-access-data) or contact them by email (globalflourishing@cos.io). All code for the analyses are openly available at the Open Science Framework (https://osf.io/wj2pk/?view_only=63ec840fc0d64211b03c57fa6d14ace7). The data from the GWP is available from Gallup, but restrictions apply to the availability of these data.

## 4 Results

Demographics, personality and social relationship quality strongly predicted WB in Sweden. We present four machine-learning models in Table 2 showcasing this. The first model evaluated 13 demographic characteristics of the participants, where several variables were dummy-coded, resulting in 55 unique predictors. These demographics predicted WB at strong levels in both the cross-validated training at *r* = .45 (*p* < .001) and generalized well to the holdout (*r* = .46; *p* < .001). We then evaluated the internal traits of the participants, i.e., their Big Five personality traits. Using the Big Five personality traits as the predictors increased the correlation with WB to *r* = .62 (*r* = .62 in the holdout; both *p* < .001). Next, we evaluated the role of the participants’ experienced social relationship quality on the regression, and these increased the cross-sectional accuracy further, up to *r* = .70 (*r* = .71 in the holdout; both *p* < .001). Lastly, we added all 65 demographic, personality traits and social relationship quality variables together. These reached an accuracy of *r* = .79 (*r* = .79 in the holdout; both *p* < .001). This level of accuracy is beyond typical acceptance levels of convergent validity (i.e., *r* = .70; Mokkink et al., 2018) and corresponds to over 63% explained variance of WB. In the supplemental material, we present pairwise combinations of the predictor variable groups (e.g., demographics and personality; Table S2).

### 4.1 Neuroticism, social relationships, and age related most strongly with Swedish well-being

A feature of Lasso regression (the machine learning algorithm used for the models in Table 2) is that it pushes redundant predictor variable's coefficients to 0 such that they do not contribute to the final model. It should be noted that just because a variable is excluded, it does not mean it does not have a relationship, independently, with WB, but rather it does not provide a contribution in the context of the other predictor variables in capturing WB. In our final WB model, 36 out of the 65 predictor variables did not contribute to the Lasso model and subsequently got a beta coefficient of 0, leaving 29 predictors contributing to the model (Fig. 3). This means the optimal model needed less than half of the predictors.

While our final model utilized a penalty of .01 optimized for WB fit on out-of-sample data (i.e. during the nested cross validation; see methods), if one utilized the next penalty in order of magnitude, .1, then another 21 variables would have been excluded. This model would only use 8 predictors (the bold predictors in Fig. 3) with only a marginal decrease on overall accuracy (from *r* = .79 to .78). Out of the eight remaining predictor variables with a beta coefficient above or below 0 after the penalty increase, three had a beta coefficient weaker than (−).10 (using the non-increased penalty). These were *extraversion* (*β* = .06), having *people to help in need* (*β* = .06), and *conscientiousness* (*β* = .05), all as positive predictors. They contributed both to the final model and the model with increased penalty, but had small effects.

The predictor variables with a beta coefficient of at least .10 were *age* (*β* = .10), *friendship contentment* (*β* = .11), and *relationship satisfaction* (*β* = .17) in the positive direction. The predictor variables with a beta coefficient below −.10, i.e., negatively related to WB, were *loneliness* (*β* = −.24) and *neuroticism* (*β* = −33). In sum, the Lasso regression predicting WB in Sweden based on demography, personality, and social relationships was primarily driven by neuroticism, loneliness, and social relationship quality. Demographic variables drove the model only to a small degree, where the only substantial predictor was age.

### 4.2 Characteristics of high and low predicted well-being in Sweden

To better understand what drove the model's high performance (from Table 2), we present illustrative cases of participants who the model predicted would have the highest and lowest WB (Table 3). Regarding demographics, the participants with the highest predicted WB tended to be older, identify with big parties, have a high income, live in large cities, and own their homes. However, regarding the psychological variables with the highest beta values (in Fig. 3) from the strongest performing model in Table 2, the participants with the highest predicted WB were very coherent, with all having maximum positive scores on relationship quality scores, and close to all having maximum negative scores on neuroticism and loneliness.

Looking at the participants with the lowest predicted WB, their demographic characteristics were young to middle-aged with low incomes. They were spread in other demographic characteristics, but they aligned strongly in their level on the psychological variables, with nearly all having maximum negative relationship quality scores and maximum scores on loneliness and neuroticism. The table highlights that relationship quality and neuroticism were the strong associates of Swedish WB and that the demographic variables mostly mattered less (giving face validity to the results of Fig. 3). Among the demographic variables, income and, in particular, age had the strongest associations.

### 4.3 Young swedes have lower well-being than older

When considering the 65 predictor variables next to each other, the fifth strongest predictor of Swedish WB, and the strongest and only substantial demographic predictor, was age. Thus, we went into more detail regarding this relationship and investigated the relationship between age and WB, split by gender (Fig. 4). First, the weighted correlation between age and WB in Sweden was positive and strong (*r* = .32, *p* < .001). Second, for both men and women, there was a slope upwards as participants were older in overall and positive WB (green [*r* = .29 for men and .36 for women] and blue lines [*r* = .27 for men and .33 for women], respectively in Fig. 4) and a slope downwards in negative WB (red lines [*r* = −.26 for men and −.33 for women]). For the “other” gender category (*n* = 33), the correlations between WB and age were in the same direction but much weaker and non-significant (all absolute *r*s < .15; all *p*s > .05). Although the correlation coefficients for men and women were similar, women's WB starting position at age 18 was considerably lower than that of men at the same age and women 10 years older. There was a particularly steep slope for women between 18 and 30, where the cohen's *d* between men and women was medium-strong (*d* = .55). After 30, the trends were similar for men and women, where participants reported a gradually higher WB with age and the higher WB for men was negligible in the 30+ sample (*d* = .10; *p* < .01). When turning to the non-binary gender, the sample was too small to compare across age groups, but comparing them towards men and women overall, they had much lower WB scores (hedges *g* = −1.43; *p* < .01).

To get a clear effect size on the age–WB relationship, we compared the average WB of participants aged 18-23 with those aged 75-80 (respectively the youngest and oldest categories in the sample). The standardised difference for men was almost a full standard deviation (*d* = .97); for women, it was over one standard deviation (*d* = 1.28). To complement these analyses, we correlated age with the three strongest predictors of Swedish WB, where loneliness had the strongest relationship to age (*r* = −.28; *p* < .001), followed by relationship satisfaction (*r* = .20; *p* < .001) and lastly neuroticism (*r* = .17; *p* < .001). In sum, there was a strong age association with Swedish WB and among the strongest WB predictors, loneliness had the strongest relationship with age.

### 4.4 The age association with evaluative well-being in Sweden started in the past years

**Fig. 5:**
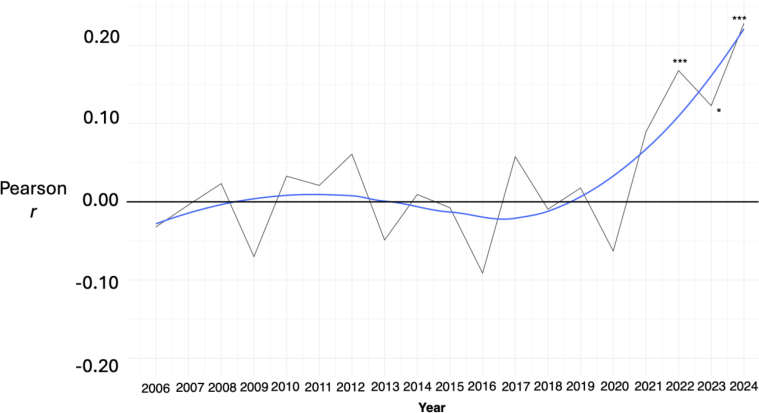
Correlation between age and life evaluation in Sweden 2006–2024

Has there always been an age association with Swedish WB? Using a different sample, the GWP data, with representative data on the Swedish population every year from 2006 to 2024, we confirmed the age association with evaluative WB in Sweden as of 2024 (Fig. 5). The WB metric in the GWP is life evaluation, which was also included in the composite WB score (Fig. 1). The relationship between age and life evaluation in the GFS data was *r* = .25 (*p* < .001) as of January-March 2023. The correlation between age and life evaluation in the GWP data as of July 2023 was *r* = .12 (*p* < .05) and *r* = .17 (*p* < .001) as of July 2022. The most recent GWP data in August 2024 showed a record high life evaluation–age relationship in the longitudinal data, reaching *r* = .23 (*p* < .001). Thus, the age–WB relationship was shown in 2022-2024 with four different representative samples.

However, the association seems to have started recently, possibly as recently as 2021. From 2006 to 2020, the average age–life evaluation relationship was more or less zero (*r*^*avg*^ = −.001; *SD* = .05). On the other hand, the age–life evaluation relationship in 2021 rose to the highest correlation thus far (*r* = .09, *p* > .05). In 2022–2024, the average correlation rose even further, up to *r* = .17 and significant levels (*p* < .05 all years). The change was driven by those below 30 getting a lower WB and those above 60 getting a higher WB (see Supplemental Material Fig. S18). In sum, we confirmed the age–WB relationship in Sweden using a different sample with one of the WB metrics in the GFS data and showed that the relationship seems to have started in recent years.

### 4.5 Individuals voting at outer edge parties generally had lower well-being

Next, we evaluated the WB of Swedes depending on which political party they identified with. It should be emphasized that political party identity explained little to no unique variance in WB next to relationship quality, personality and other demographic information (Fig. 3). Still, political party identity significantly predicted WB without the other predictor variables in a Lasso regression (*r* = .18, *p* < .001). Thus, while the non-zero predictor variables in Fig. 3 (e.g., loneliness and neuroticism) explained nearly all variance in WB that political party identity did, WB levels still differed significantly among political party identities.

**Fig. 6:**
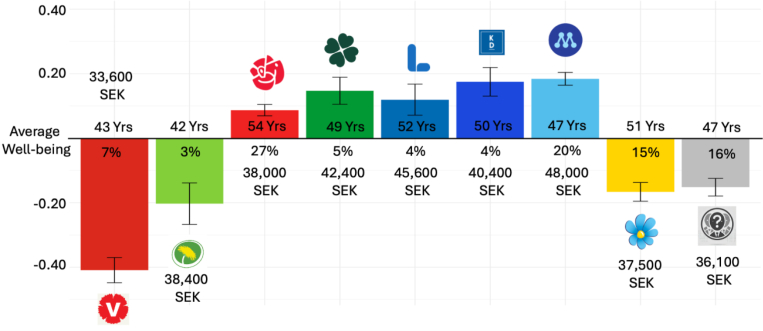
Well-Being and political party in Sweden

In Fig. 6, we visualize how the WB differed for different political party identities, along with the percentage of participants identifying with each party and their average age and household income (the two strongest demographic predictors of Swedish WB). Generally, the parties commonly associated as “outer edge parties” and respondents not identifying with any party had a lower WB than the average Swede (−.41, −.20, −.17, and −.15 standardised units below the Swedish WB mean for The Left Party, The Green Party, The Swedish Democrats, and respondents with no political party identity, respectively). On the other hand, the classically social (The Social Democrats), central (The Centre Party and The Liberals), and conservative (The Christian Democrats and The Moderates) parties all had average WB above the Swedish average. The strongest WB difference was between The Moderates and The Left Party (standardised mean difference = .59, Cohen's *d* = .67, *p* < .001).

In general, all classical bourgeois parties (The Moderates, The Christian Democrats, The Liberals, and The Centre Party) had average monthly incomes of 40,000 SEK and above. In contrast, the left parties (The Left Party, The Green Party and the Social Democrats) and The Swedish Democrats had less than 39,000 SEK in monthly household income. The two most left parties in Swedish politics generally had the youngest voters (43 and 42 years for The Left Party and The Green Party, respectively), and The Social Democrats had the highest mean age (54 years).

To explain the WB map, we utilized the latest expert ratings on political party opinions in Europe (Rovny et al., 2025) to factor analyze 50 items experts rated nearly all European parties on, and used the two first factors to map the Swedish political parties. The first factor mimicked the GAL-TAN scale (i.e., Green, Alternative and Libertarian on the one end to Traditional Authoritarian and Nationalistic on the other hand) and explained 42% of the variance. The second component mimicked classic economic left to right and explained an additional 14% variance. When adding the GAL-TAN scale on the x-axis and economic left to right on the y-axis (left in the bottom), the two-dimensional figure looked similar to Fig. 3 below (see Supplemental Material Fig. S19). That is, The Left Party, The Green Party, and The Swedish Democrats were similarly economically left and in the bottom while The Left and Green Parties were far from the Swedish Democrats on the GAL-TAN scale. The remaining parties were more in the middle and high.

### 4.6 Differences in well-being based on multiple demographic variables in Sweden

In the supplemental material, we show differences in WB scores for several demographic variables and their subgroups. For example, they include regional differences (Fig. S1), where Halland was the region with the highest WB and Västmanland the region with the lowest WB. The data also show that married Swedes have the highest WB among marital statuses in Sweden and singles the lowest (Fig. S2), that unemployed Swedes have the lowest WB among employment statuses and retired and self-employed the highest (Fig. S4), and that Swedish Christians have a higher WB than Swedish atheists and Muslims (Fig. S8). The data also include WB differences based on whether someone is born in Sweden or abroad (Fig. S3), their education level (Fig. S5), whether they live in a rural or urban setting (Fig. S6), whether they live alone vs with others (Fig. S7), differences in spirituality (Fig. S9), and in religious service attendance (Fig. S10). Overall, these demographics had small effects on WB compared to the psychological variables and age, but may be of interest to the Swedish public.

Lastly, the supplemental material includes a Lasso model that included all variables that had a correlation with WB above *r* = .30 (which can be seen in Fig. S11). By doing that, we predicted WB at *r* = .87 (see Table S2). Physical health, optimism, freedom, gratitude and incapability of doing things in life were meaningful predictors that added to the accuracy (Fig. S14). Still, neuroticism and loneliness were the strongest predictors.

## 5 Discussion

The present analysis constitutes the first WB study with large representative data solely focused on Sweden and the psychological predictors of Swedish WB. The study showed that: i) demographics, personality and social relationships predicted Swedish WB strongly (*r* = .79); ii) age, neuroticism, and loneliness were the strongest predictors of WB among demographics, personality traits, and social relationships, respectively; iii) age is a medium to strong and quite new predictor of Swedish WB; and iv) while age was the only close-to substantial demographic predictors for our machine learning model next to personality and social relationships, meaningful differences within them existed, such as for political identity.

That demographics, personality, and social relationships predict Swedish WB is not surprising, considering they are all established predictors of WB (e.g., Anglim et al., 2020; Hansson et al., 2005; Quoidbach et al., 2019). However, personality explains less than 50% of the WB variance by itself (Anglim et al., 2020), and there is little meta-analytic evidence on how much variance social relationships and demographics typically explain in WB. Our results show that combining the three domains explained 65% of the variance in WB,^3^ which, in almost any psychological context, is a large amount (Gignac & Szodorai, 2016; Paterson et al., 2016; Mokkink et al., 2018).

Age was the strongest demographic WB predictor in the machine learning model, with older Swedes having higher WB than young ones. Since age turned out to be such a strong predictor, we confirmed the relationship with complementary GWP data, harnessing the same evaluative WB metric (the Cantril Ladder) that is used in the World Happiness Report. While the age–evaluative WB relationship was weaker in the GWP than in the GFS data (*r* = .15 v. *r* = .25, using the same life evaluation metric), the GWP data also suggested that the positive and significant relationship only started fairly recently. Previous research has shown a zero relationship between age and WB in Sweden cross-sectionally and longitudinally, from 1982 to 2009 (Hansson et al., 2005; Realo & Dobewall, 2011). The GWP data also supported this zero relationship until 2009, continuing up to 2020, when the age–evaluative WB association started, and that the change was driven both by the young getting a lower WB and an increase in older Swedes' WB (Fig. S18). Particularly alarming is the considerably low WB among young Swedish women. Although gender did not have a contributing effect in the regression model, young women's WB was considerably lower than young men's, and the age association with WB was slightly stronger on women than on men. Additionally, the few 33 participants with another gender than male or female scored much lower on WB in comparison to men and women.

Our findings align with other emerging research showing that the WB among youth in parts of the world, especially in high-developed countries, has declined in recent years (Buecker et al., 2021; Helliwell et al., 2024), including in Sweden (Statistics Sweden, 2023b; Helliwell et al., 2024). One prominent candidate often invoked to explain this change is the introduction of smartphones and social media from around 2010; those who were in their early teens at that point would then reach adulthood–and participate in surveys like the GWP–around 2015, just as the trend seemed to begin in Sweden. Among young people in Sweden, almost everyone uses smartphones and social media daily (The Swedish Internet Foundation, 2024), and screen time has been shown to have a longitudinal impact on both sleep quality and either directly or indirectly on depression levels (Hökby et al., 2025). Based on such findings, the Swedish government has proclaimed that “Children's screen use is one of the biggest health challenges of our time.” (*p* 1. Government Offices of Sweden, 2025) and in October 2025, the Swedish Public Health Agency introduced guidelines on screen time for children and young people in Sweden to tackle the challenge, on behalf of the government (Public Health Agency Folkhälsomyndigheten, 2025a). At the same time, neighboring Denmark recently reached parliament consensus to introduce an age limit of 15 years on social media to tackle the challenge (The Danish Ministry of Digitalisation, 2025). However, while smartphones and social media are strong candidates for the rising age–WB relationship, as suggested by increased loneliness effects of social media usage in younger individuals outside of Sweden (Luhmann et al., 2023; Nowland et al., 2018), there is not yet conclusive evidence for the hypothesis, though evidence continues to accumulate (Capraro et al., 2025).

Simultaneously, just as screen time, smartphones, and social media have increasingly become a natural part of everyday life in Sweden, conversely participation in physical and sport activities have decreased (Gothilander, Almqvist, Eriksson, & Fritz, 2024; Kägi-Braun et al., 2025). Sports and physical activities have shown strong effects for both mental health aspects such as self-esteem and relationship qualities such as prosocial competence among Swedish young people (Wagnsson et al., 2013), and so the decline in these behaviours may also be a factor in the decline in youth WB.

The data suggested that individuals identifying with political parties in the outer edge of the political spectrum, or who did not have any party to identify with, had the lowest WB. When political identity has been measured on a continuum from liberal to conservative, conservative individuals have typically had a higher WB than liberals (Napier & Jost, 2008; Okulicz-Kozaryn et al., 2014; Stavrova & Luhmann, 2016). However, the key distinction in Sweden seems to have been how far parties are from the middle and whether they were economically liberal, where economically liberal parties in the middle had a higher WB, while outer edge parties that are less economically liberal have a lower WB (Fig. S19). When we looked at hope for the future, the Left Party and the Green Party had a decrease compared to the Swedish Democrats (compared to just WB), perhaps stemming from the fact that the Swedish Democrats were closer to government power in 2023 (when the data was collected). In future, we will be able to harness both GFS data to see if WB changes in relation to governmental dynamics (e.g., if there is a government shift in the next election).

Regarding personality, it was mainly neuroticism that contributed with predictive accuracy to the Lasso model, and this was the strongest overall predictor next to social relationships and demography. While this finding was expected, more surprising was that extraversion and conscientiousness had a relatively low association with WB besides demographics and relationships (Anglim et al., 2020). Since extraversion is a trait primarily focused on human interaction, it is not surprising that the social relationship variables would capture the same variance as extraversion and beyond. However, it is more challenging to explain why conscientiousness turned out to be such a weak predictor compared to the other variables. Among the variables that had a stronger contribution to our WB model than conscientiousness, age had the strongest correlation with conscientiousness (*r* = .25). Thus, age might have captured parts of the conscientiousness–WB variance, and indeed conscientiousness typically rises with age although it starts to decline around 50 (Bleidorn et al., 2022).

That social relationships would predict WB strongly was expected (Proulx et al., 2007; Quoidbach et al., 2019). The strongest social relationship-related predictor was loneliness, which has previously been shown to have a strong negative association with WB (Park et al., 2020), and even physical health, for which it appears to be as bad as smoking (Pantell et al., 2013). Also the positively anchored relationship variables relationship satisfaction, contentment with friends and having people to help in need all contributed to the machine learning model, indicating that the variance explained by social relationships was spread out across 4 out of the 5 social relationship variables.

### 5.1 Implications for Swedish politicians and public policy

This study offers several potential implications for Swedish civil society, politicians and public policy makers, the most vital being young Swedes' considerably lower WB and higher loneliness compared to older Swedes, especially among young women. There is evidence that Swedish youth unhappiness and loneliness is a trend starting only recently, with the age–WB relationship being near zero between 1982 and 2020 (Hansson et al., 2005; Realo & Dobewall, 2011, Fig. 5). Further, loneliness is typically not linearly related to age in other studies and contexts (Hawkley, Buecker, Kaiser, & Luhmann, 2022; Mund et al., 2020) and if it is linear, loneliness is typically thought to increase with age (Surkalim et al., 2022), including in Sweden (Dahlberg et al., 2015). However, our data showed the opposite, which shows loneliness *decreasing* with age. Overall, the age effect in Sweden is to some degree unexpected and may have emerged recently. Politicians and public policy makers should be aware of these changes–both in terms of the lower WB of youth, and also the high level of WB among older Swedes, particularly retirees. The latter finding may be an effect of Sweden's welfare system, where pensionaries often have autonomy in their lives for a long time and do not have to rely on family members to take care of them when being ill.

Apart from loneliness, the remaining social relationship variables (e.g., relationship satisfaction) were also substantial predictors of WB. If Sweden is going to prioritise WB in its population and in particular in its youth, nurturing social relationships should be of key interest. To support this, a recent meta-analysis on pre-registered experiments on increasing WB showed that few popular WB interventions reliably increased WB (Folk & Dunn, 2024). However, being more sociable reliably increased WB. Positively, the Swedish institutions have recognised the loneliness problems as an increasingly recognised issue, where the Public Health Agency of Sweden recently published the strategy “Standing together: A national strategy to tackle loneliness” (Public Health Agency, 2025b). They address three core themes of the strategy, i) learning about the impact of loneliness, ii) raise awareness of the impact of loneliness, and iii) working together with different stakeholders to find solutions. While public policy may be able to do more, the media, schools, and broader civil society should also make efforts to work with these strategies. Research like the present study may help in addressing the first two themes, but need help from other parts of society, including media and schools. In parallel with the loneliness strategy, the recommendations on screen time for young people (Public Health Agency Folkhälsomyndigheten, 2025a) and increased physical activities in groups, may be other paths forward to deal with the loneliness and social relationships problems in Sweden, particularly among the young (Gothilander et al., 2024; Kägi-Braun et al., 2025; Wagnsson et al., 2013).

Personality, and particularly neuroticism, was the strongest predictor of WB. While the age and loneliness relationships to WB were unexpected and it is possible to some extent to act to address this, with neuroticism this is typically a stable trait that is highly driven by genetics (Nes & Røysamb, 2017). That portions of individuals' WB are stable is important for politicians to know since WB cannot be fully altered. Thus, if Swedish politicians should focus on anything to increase Swedish WB, it is to understand the increased age association with Swedish WB (which is not typical in the world's societies; Helliwell et al., 2024), prioritise the youth and work towards a society with increased social relationship quality.

### 5.2 Limitations

The data have several limitations which need acknowledging. First, the data was non-probabilistically sampled but included weights for each participant based on official Swedish statistics. The data is roughly nationally representative, with Gallup being among the most experienced organizations in collecting representative data. However, the data can be seen as proportional to a representative sample due to the non-probabilistically based sampling with quotas based on age, gender, region, and educational level (Ritter et al., 2024, p. 12). In particular, older Swedes with poor digital literacy may have been excluded. However, Gallup estimated that less than 2% of the population was excluded due to the recruitment approach (Ritter et al., 2024). Second, the GFS data reported here is cross-sectional and while the GWP data runs longitudinally, it is not experimental. Thus, the results should not be interpreted causally. Third, the data exclusively uses self-reports and is thus exposed to common method variance, a large problem in social science (Podsakoff et al., 2024), which likely inflated the accuracy of predicting WB. For example, social desirability has explained around half of the variance that personality and WB share (Persson et al., 2025). On the other hand, many of the measures were brief, including the personality scales, and disattenuating the relationship to WB would increase the relationship between the predictors and WB, potentially compensating for the inflated relationship due to common method variance.

While the outcome encompassed a broad range of WB dimensions, further insights could have been gained by analyzing individual components of the composite WB measure. However, to include multiple dimensions of WB, without exhausting the paper, we decided to focus on composite WB. Further, since the internal reliability of the composite score was very high (e.g., Cronbach's Alpha of .94), the composite score likely captured a common WB factor. Important recent work has focused on different WB dimensions and their characteristics in the Swedish population and also demonstrated the age–WB association in Sweden (Bittár et al., 2025). In a similar vein, future research could examine what predictors are important for SWB in Sweden when breaking down demographic characteristics, such as how important personality and relationships are for various age groups or genders (see Schaefer et al., 2024).

An aspect of the Lasso regression is that just because predictors were given a beta coefficient of 0, it does not mean that their relationship with WB was necessarily 0. The bivariate correlations (Fig. S12) can be used to examine the individual relationships. The beta coefficients from the Lasso regressions should be interpreted such that when the specific predictor variables are added next to each other, the non-zero beta coefficients are the predictors that were meaningful to the Lasso regression model.

Although we replicated the correlation between life evaluation and age in the GWP data, the strengths differed, ranging from *r* = .12 for the GWP data in July 2023, to *r* = .25 in the GFS data in January-March 2023, and *r* = .17 in the GWP data July 2023. While we can not establish the reason for this difference despite nationally representative samples, potential explanations could be i) the time of the year of the data collections (winter for the GFS, summer for GWP) or ii) the different sample sizes (around 1000 in GWP vs 15,000 in the GFS data).

Lastly, the data is subject to cohort, period, and time interpretative complications. For the age–WB relationship, we could complement the findings with World Poll data, but not for the remaining analyses. However, the GFS is envisaged as conducting data collection over at least five years–with Wave 3 already recently released, and Wave 4 due to begin in 2026–and thus, future research will help with further interpretation.

### 5.3 Conclusion

This paper has offered the first evaluation of multidimensional Swedish WB in a large, nationally representative sample with a specific focus on demographics, personality and social relationships as predictors. Using these as predictors in a machine learning model, we explained 65% of Swedish WB, where neuroticism, loneliness, and relationship satisfaction were the strongest predictors. Beyond these three, age was the fourth strongest overall predictor–which is a relatively new finding in Sweden–which we confirmed with complementary representative data from the GWP, further indicating that the relative decline in Swedish youth WB began only recently (i.e., since 2020). Multiple additional findings regarding specific demographics and other psychological variables such as political party and optimism can by itself also describe Swedish WB. We hope that the findings will inform Swedish politicians and public policies as WB is becoming an increasingly important outcome for Swedish policy.

## CRediT authorship contribution statement

**August Håkan Nilsson:** Writing – review & editing, Writing – original draft, Visualization, Software, Methodology, Investigation, Formal analysis, Conceptualization. **Petri J. Kajonius:** Writing – review & editing, Writing – original draft, Methodology, Investigation, Conceptualization. **Oscar Kjell:** Writing – review & editing, Supervision. **Micael Dahlen:** Writing – review & editing, Validation. **H. Andrew Schwartz:** Writing – review & editing, Validation, Supervision, Methodology. **Brendan Case:** Writing – review & editing, Funding acquisition. **Byron Johnson:** Writing – review & editing, Funding acquisition. **Tim Lomas:** Writing – review & editing, Project administration, Funding acquisition, Conceptualization. **Noah Padgett:** Software, Methodology, Formal analysis, Data curation. **Tyler J. VanderWeele:** Writing – review & editing, Resources, Project administration, Funding acquisition.

## Disclosures

T.J.V. reports consulting fees from Gloo Inc., along with shared revenue received by Harvard University in its license agreement with Gloo according to the University IP policy.

## Ethics approval statement and informed consent

This project was ruled EXEMPT by the Baylor University Institutional Review Board (#1841317-2). Gallup Inc. IRB approved the study on November 16, 2021 (#2021-11-02). All data collection was performed in accordance with the ethical standards of Gallup and with the 1964 Helsinki Declaration and its later amendments. Informed consent is obtained during the respondent recruitment stage of fieldwork. Consent is obtained at the start of the survey. The exact wording varies across countries depending on the local laws and regulations governing data protection. Subsequent surveys include a consent statement that reminds respondents that participation in the survey is optional and their personal information will not be shared by anyone outside of Gallup.

## Declaration of competing interest

T.J.V reports consulting fees from Gloo Inc., along with shared revenue received by Harvard University in its license agreement with Gloo according to the University IP policy. Other authors have no conflicting interests.

## Data Availability

The authors do not have permission to share data.

## Footnotes

1 That is, higher levels of happiness occur in one's both younger and older years.

2 There were no official summary statistics on mean age for only the adult population in Sweden. But the data for the amount of persons at each age was officially available and we used this to calculate the mean adult age in Sweden.

3 When we added all variables in the data with a correlation to WB stronger than (−).30 in a final Lasso regression, optimism, physical health, freedom pushed up the WB accuracy beyond *r* = .79, explaining variance beyond demographics, personality and social relationships. Together, the data-driven model had an accuracy of r = .87, explaining 75% of Swedish WB variance, where neuroticism and loneliness remained as the strongest predictors, and age as the strongest demographic predictor.
