# Supplementary material for: Swedish Well-Being: The rising importance of age among demographic, personality, and social relationship factors
Source: SSM Popul Health. 2026 Apr 1;34:101913. doi: 10.1016/j.ssmph.2026.101913 (PMC13223817; doi:10.1016/j.ssmph.2026.101913)
Supplement: Multimedia component 1 [file mmc1.docx]

Swedish Well-Being: The Rising Importance of Age Among Demographic, Personality, and Social Relationship Factors

## **Supplemental material**

**Figure S1.** Well-Being and Region in Sweden
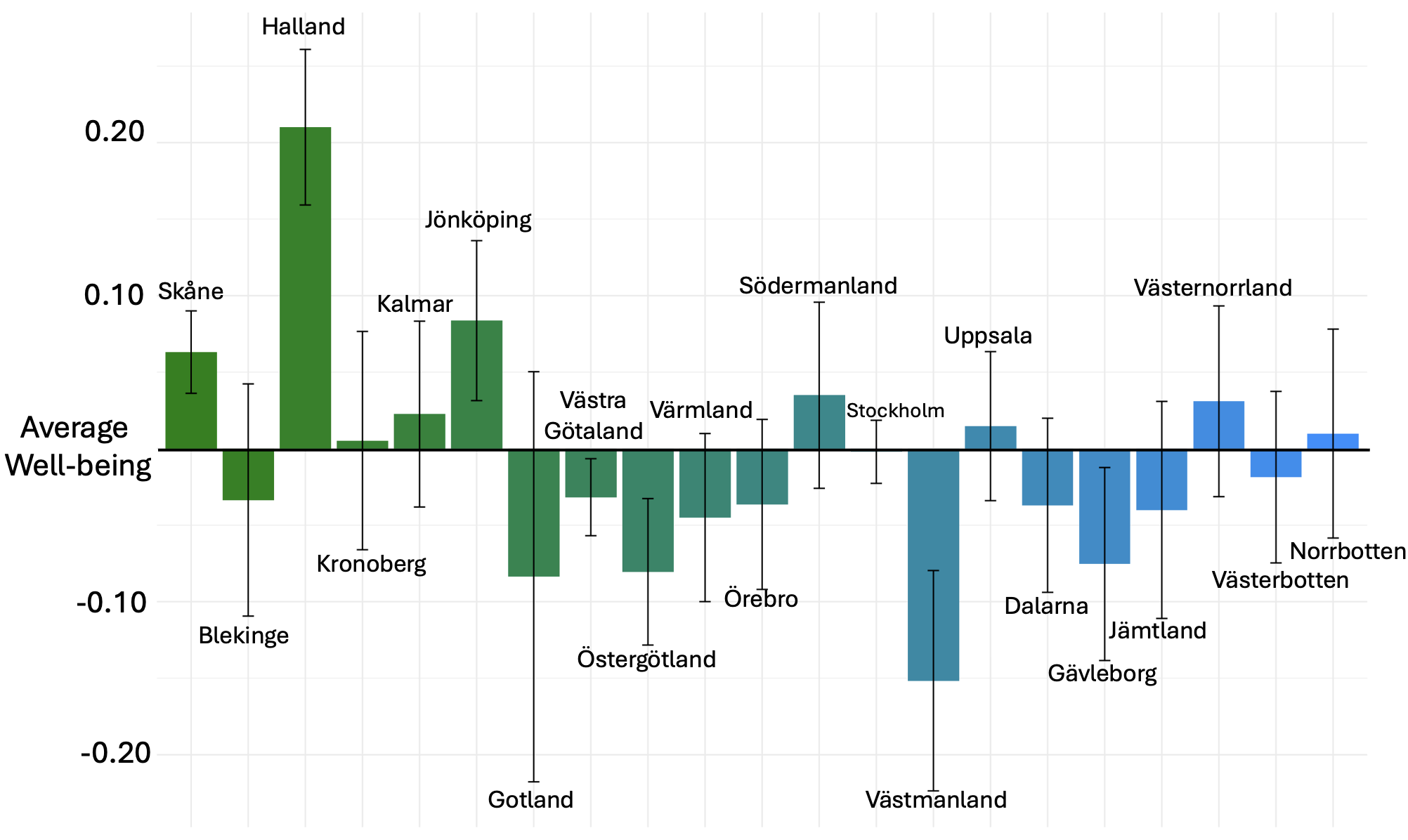


*Note*: Regional differences in SWB across Sweden. Bars indicate the average SWB scores by region, centered around the national average (zero). Error bars represent 95% confidence intervals. Regions are colored by their geographical location, with green shades indicating southern Sweden and blue shades indicating Northern Sweden.

**Figure S2.** Well-Being and marital status in Sweden

**
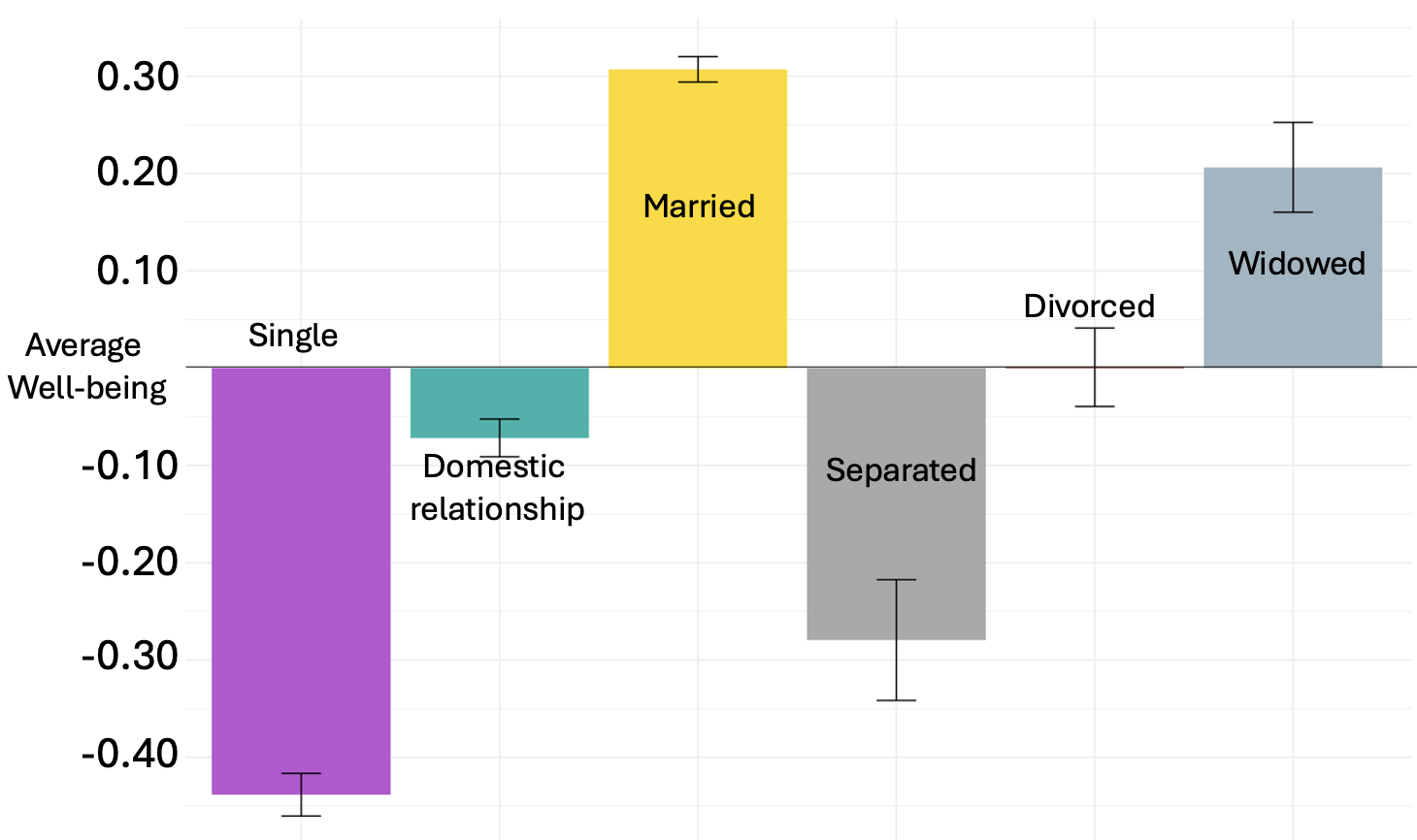
**

*Note*: Marital status differences in SWB across Sweden. Bars indicate the average SWB scores by marital status, centered around the national average (zero). Error bars represent 95% confidence intervals.

**Figure S3.** Well-Being and place of birth * gender in Sweden
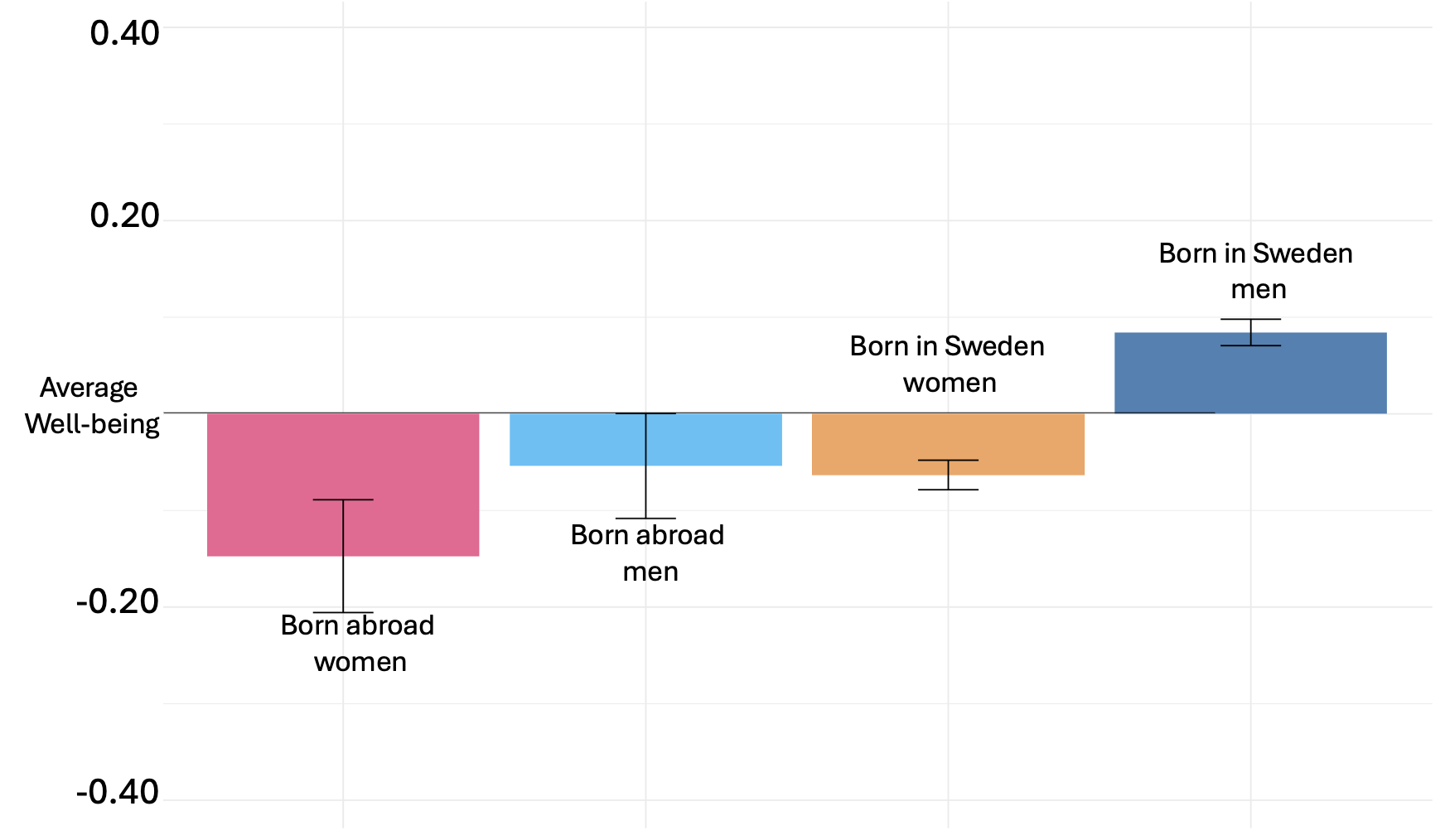


*Note*: Place of birth * gender differences in SWB across Sweden. Bars indicate the average SWB scores by place of birth * gender, centered around the national average (zero). Error bars represent 95% confidence intervals.

**Figure S4.** Well-Being and employment status in Sweden
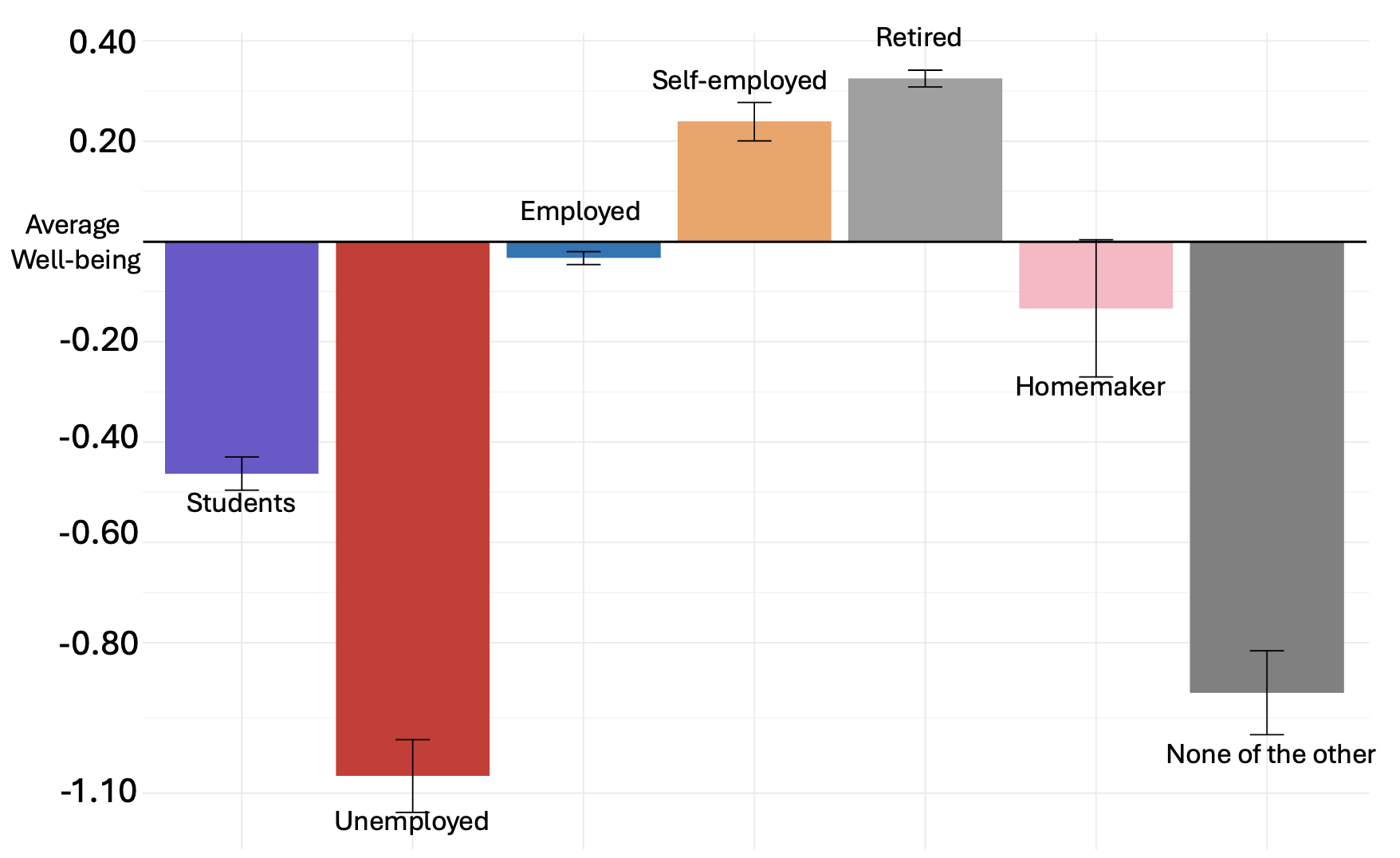


*Note*: Employment status differences in SWB across Sweden. Bars indicate the average SWB scores by marital status, centered around the national average (zero). Error bars represent 95% confidence intervals.

**Figure S5.** Well-Being and education level in Sweden

**
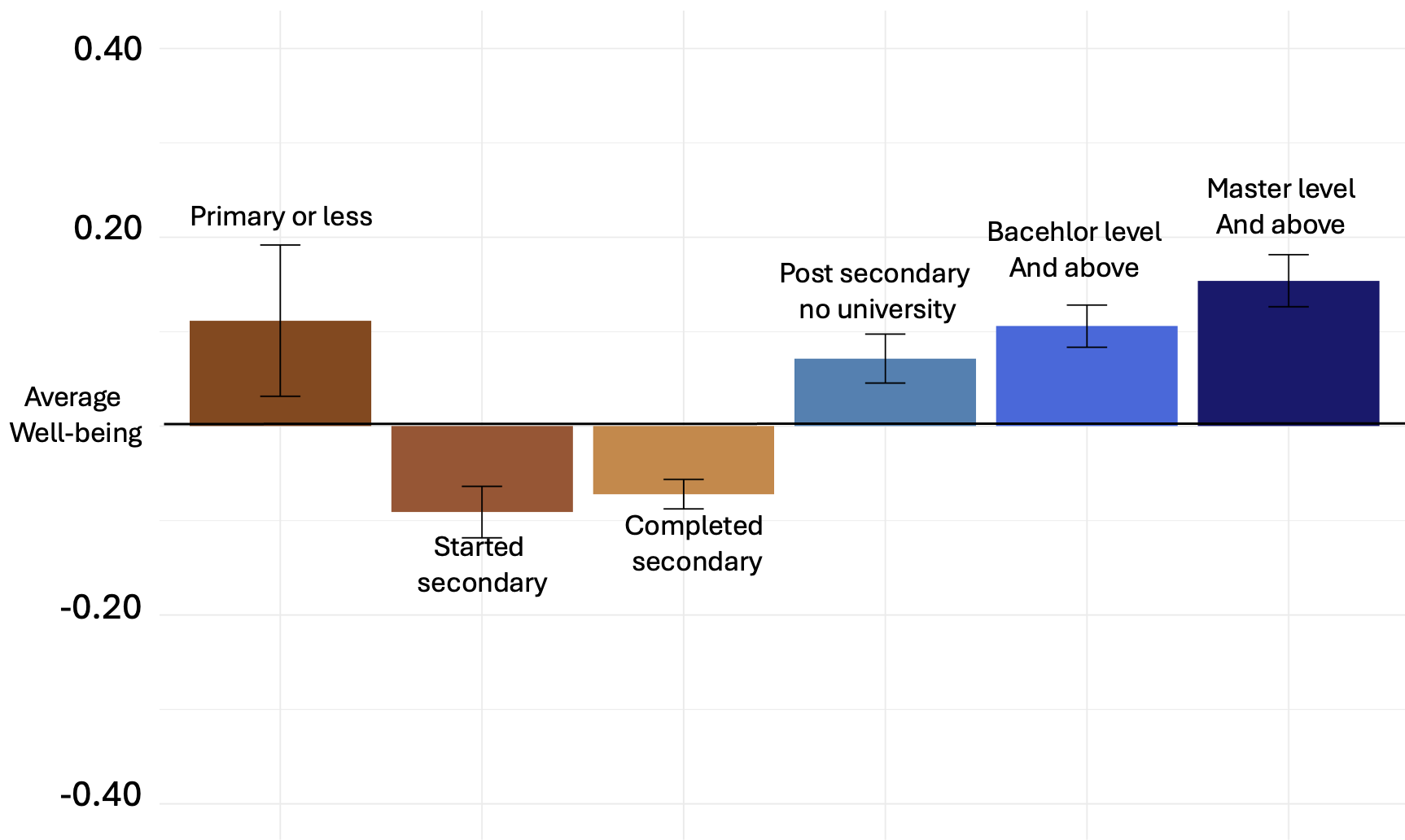
**

*Note*: Education level differences in SWB across Sweden. Bars indicate the average SWB scores by marital status, centered around the national average (zero). Error bars represent 95% confidence intervals.

**Figure S6.** Well-Being and rural vs urban living in Sweden

**
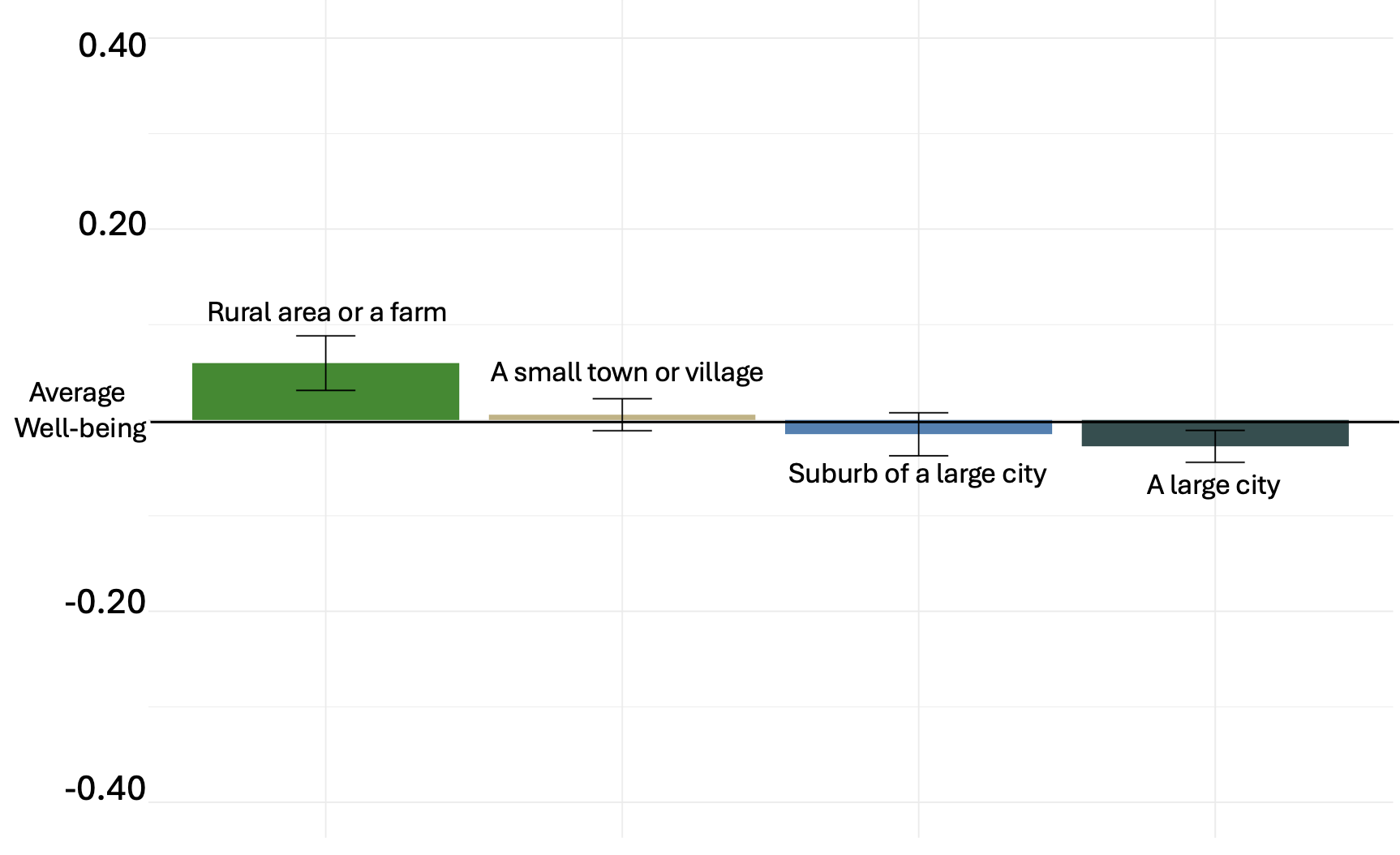
**

*Note*: Rural vs urban living differences in SWB across Sweden. Bars indicate the average SWB scores by marital status, centered around the national average (zero). Error bars represent 95% confidence intervals.

**Figure S7.** Well-Being and living alone with gender in Sweden
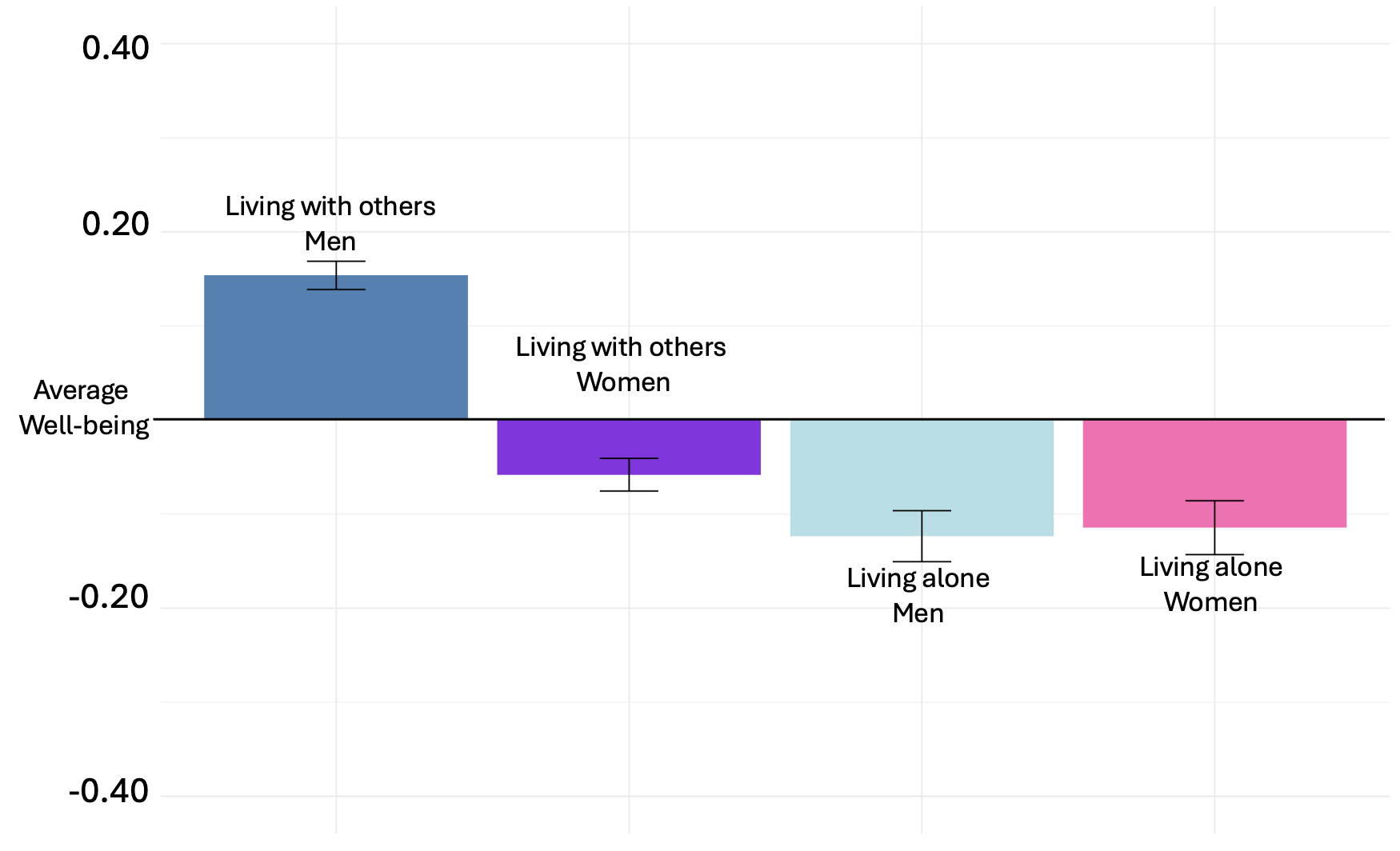


*Note*: Living alone * gender differences in SWB across Sweden. Bars indicate the average SWB scores by marital status, centered around the national average (zero). Error bars represent 95% confidence intervals.

**Figure S8.** Well-Being and religion in Sweden

**
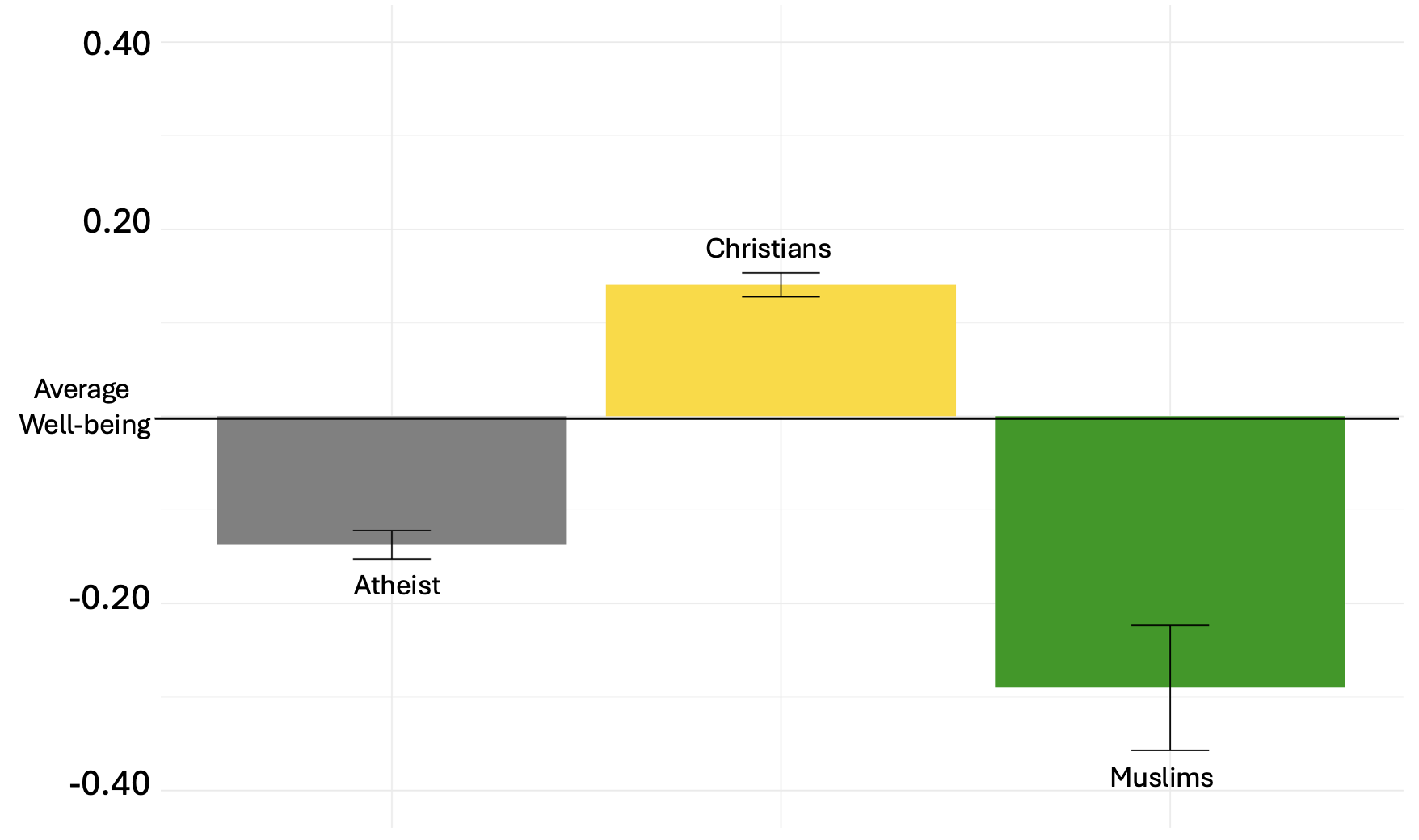
**

*Note*: Religious affiliation differences in SWB across Sweden. Bars indicate the average SWB scores by marital status, centered around the national average (zero). Error bars represent 95% confidence intervals. Other religions had very low sample sizes and were thus excluded.

**Figure S9.** Well-Being and spirituality in Sweden
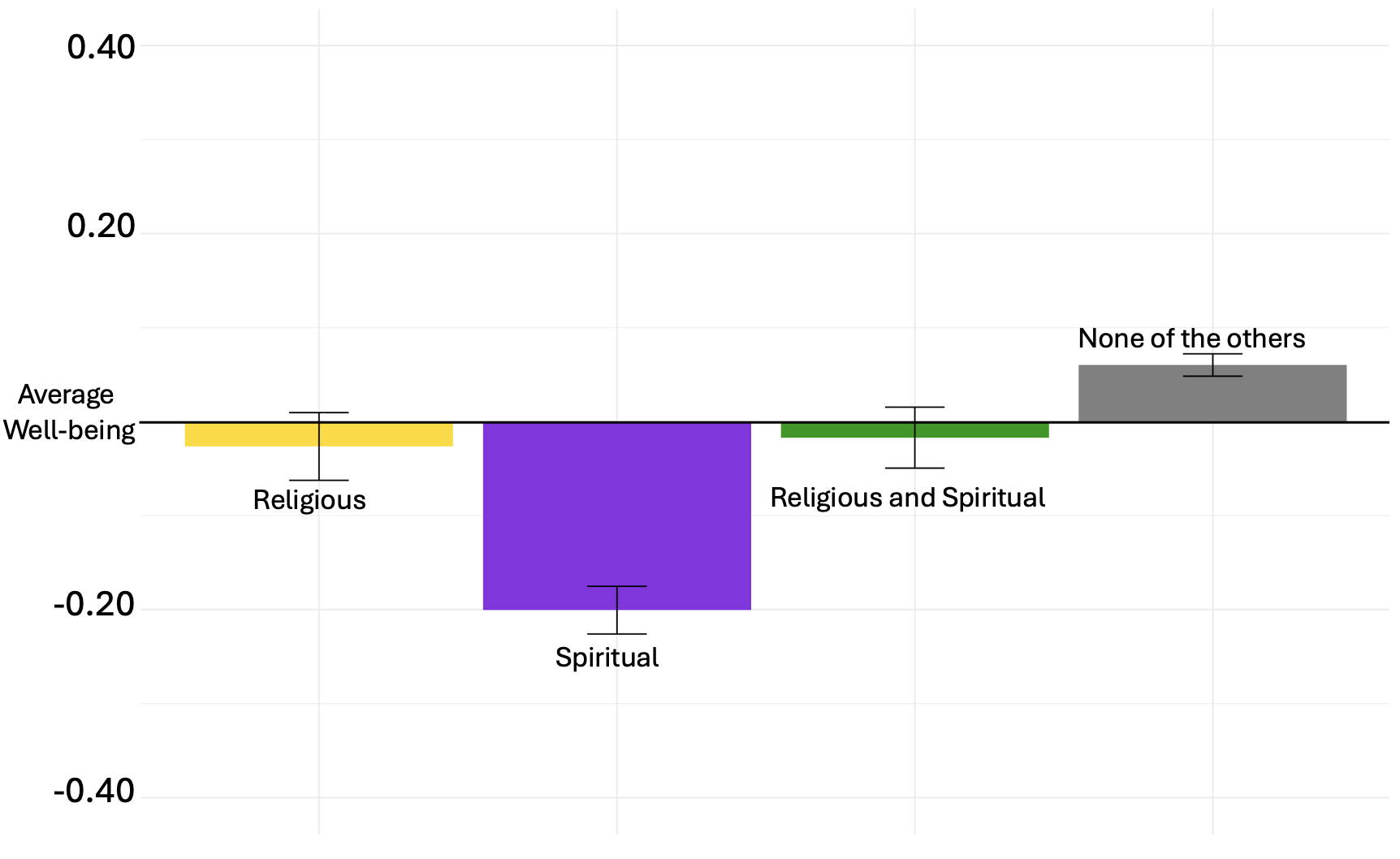


*Note*: Spirituality differences in SWB across Sweden. Bars indicate the average SWB scores by marital status, centered around the national average (zero). Error bars represent 95% confidence intervals.

**Figure S10.** Well-Being and Religious Service Attendance Frequency in Sweden
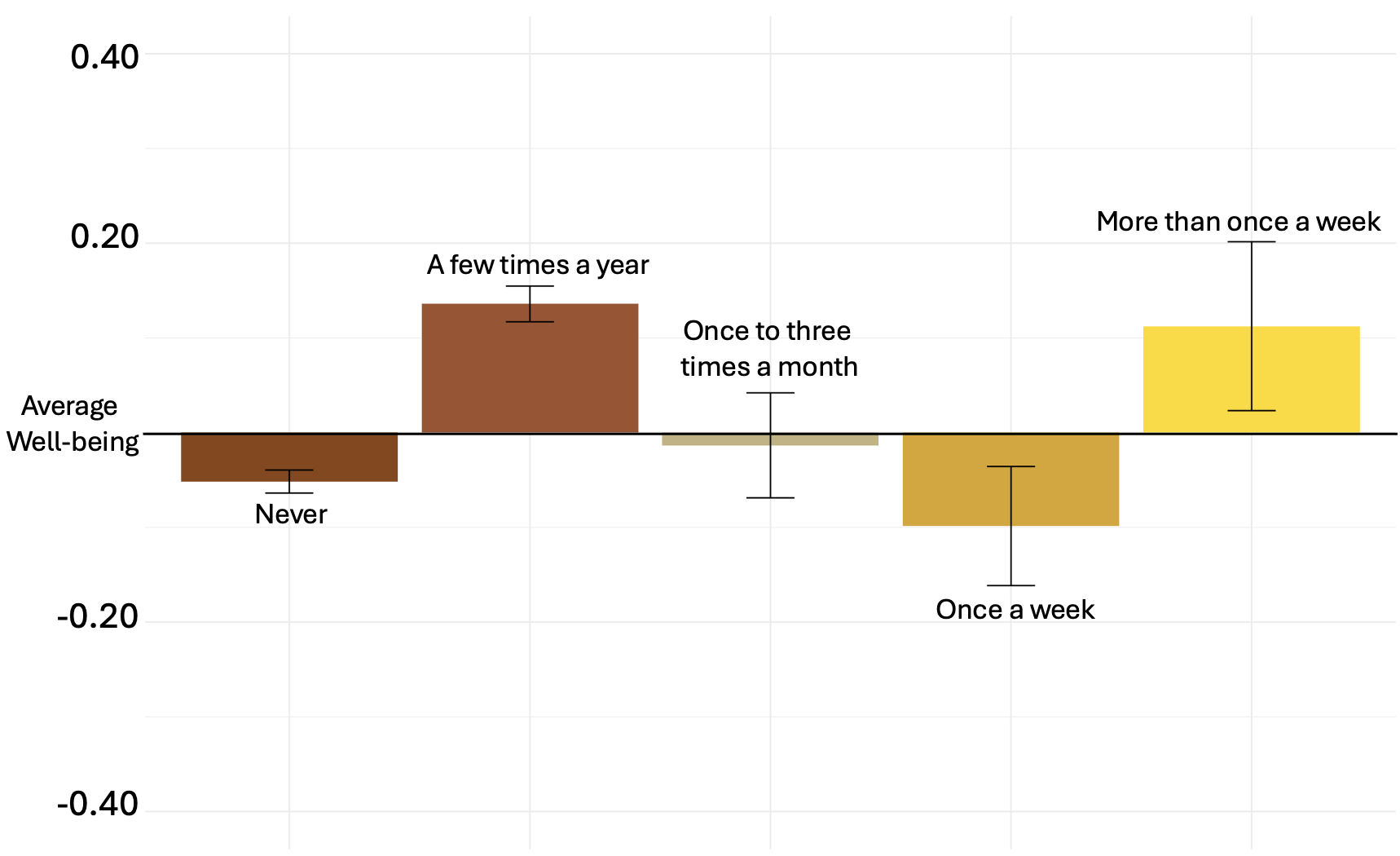


*Note*: Religious service attendance frequency differences in SWB across Sweden. Bars indicate the average SWB scores by marital status, centered around the national average (zero). Error bars represent 95% confidence intervals.

**Figure S11.** Well-Being and monthly household income in Sweden
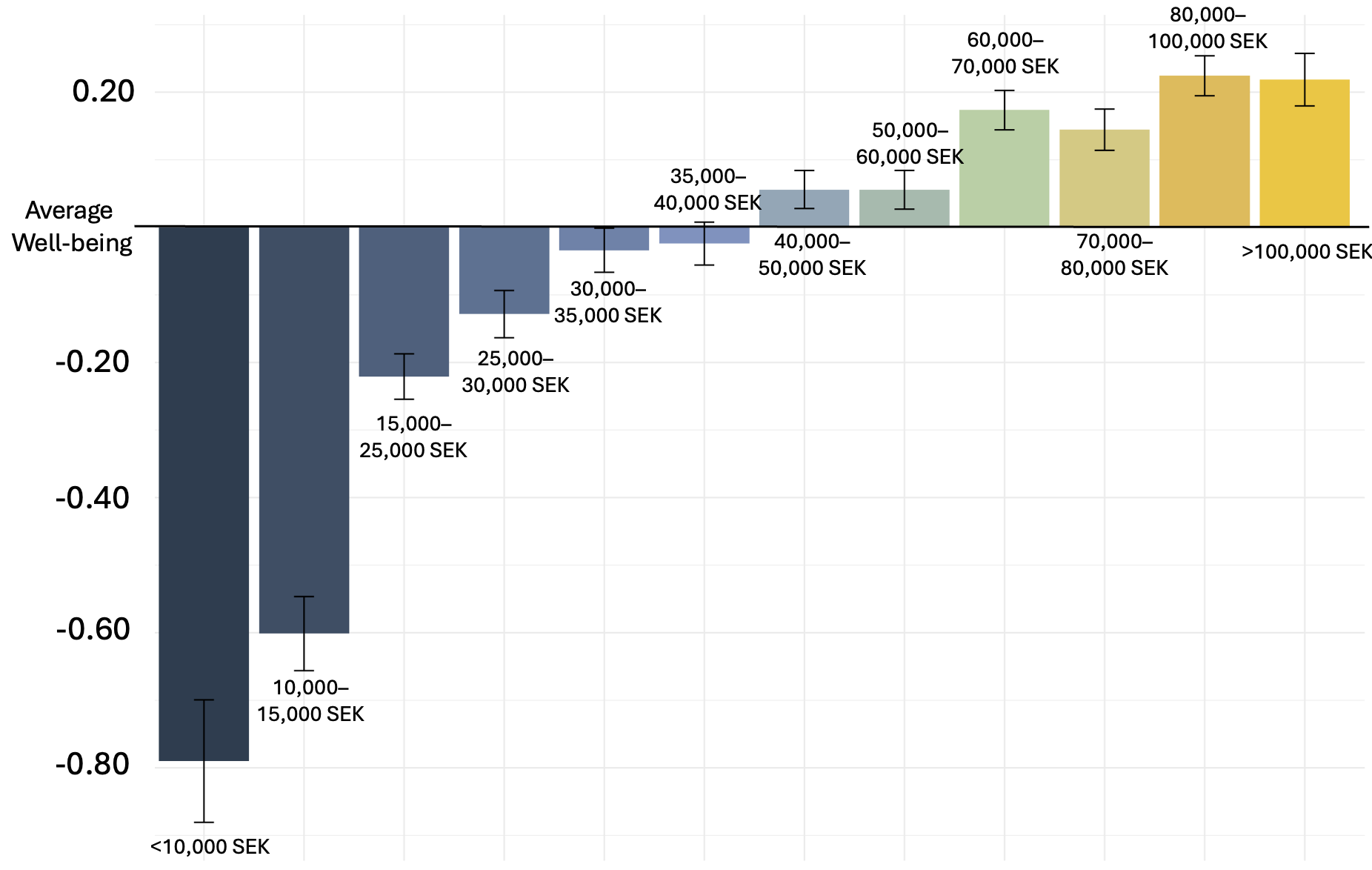


*Note*: Monthly household income differences in SWB across Sweden. Bars indicate the average SWB scores by region, centered around the national average (zero). Error bars represent 95% confidence intervals. Income groups are colored from dark blue (low income) to gold (high income).

**Figure S12.** Bivariate weighted Pearson correlations with Well-Being outcomes.**
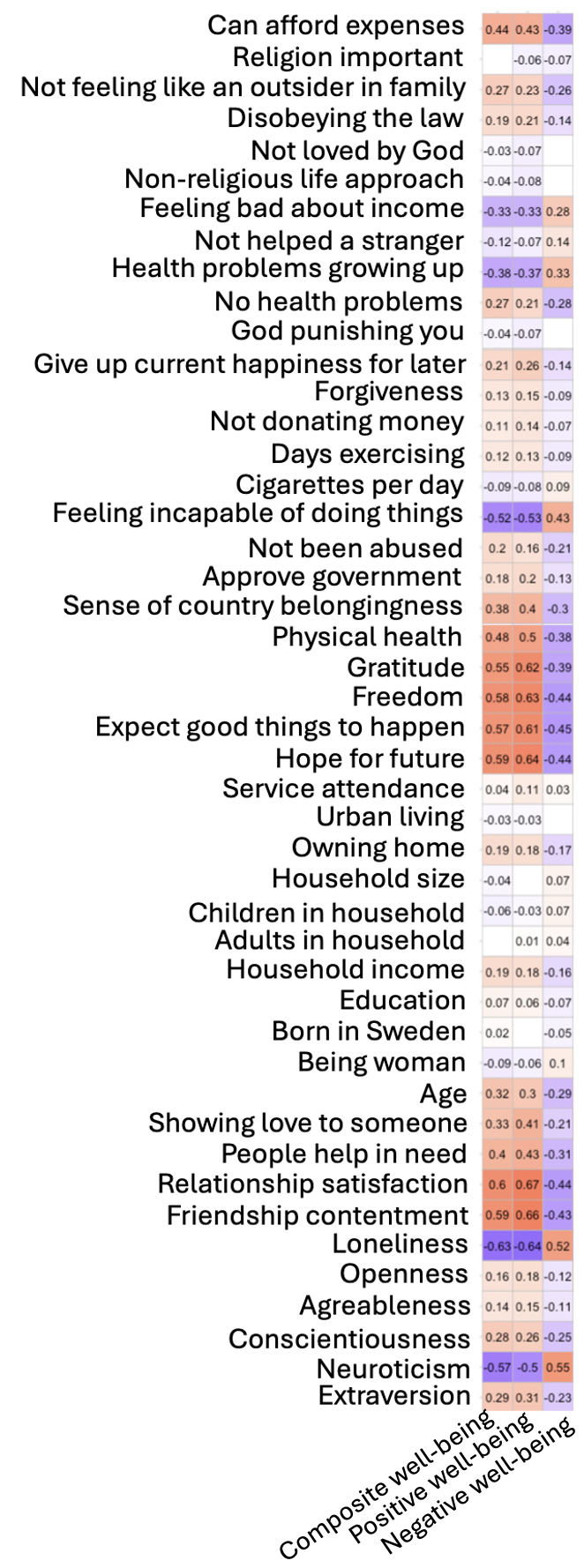
**

*Note*: White boxes were non-significant. Remaining correlations all had *p* < .05.

**Figure S13.** Bivariate correlations between SWB variables


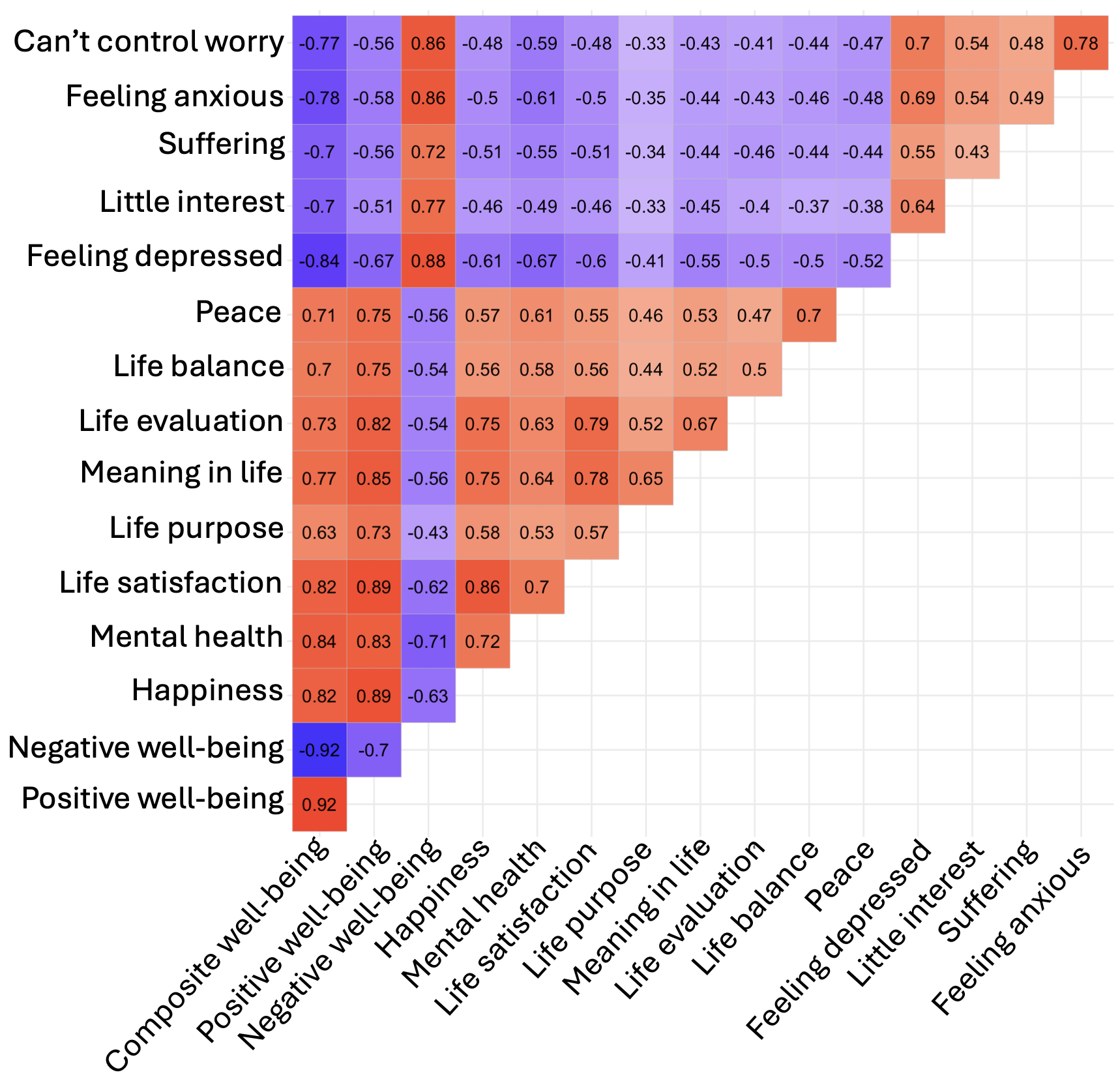


*Note*: All *p* < .001.

**Table S1.** Items for the study variables.

| **Domain & Construct** | | **Exact Survey Question / Item** | **Response Scale** |
| --- | --- | --- | --- |
| **Positive Well-Being** | |  |  |
|  | Happiness | "In general, how happy or unhappy do you usually feel?" | 0 (Extremely unhappy) to 10 (Extremely happy) |
|  | Life satisfaction | "Overall, how satisfied are you with life as a whole these days?" | 0 (Not at all satisfied with your life) to 10 (Completely satisfied) |
|  | Life evaluation today | "On which step of the ladder would you say you personally feel you stand at this time?" | 0 (Worst possible) to 10 (Best possible) |
|  | Peace | "In general, how often do you feel you are at peace with your thoughts and feelings?" | 1 (Always) to 4 (Never) *(reverse-coded)* |
|  | Balance in life | "In general, how often are the various aspects of your life in balance?" | 0 (Extremely unhappy) to 10 (Extremely happy) |
|  | Meaning | "Overall, to what extent do you feel the things you do in your life are worthwhile?" | 0 (Not at all worthwhile) to 10 (Completely worthwhile) |
|  | Purpose | "I understand my purpose in life." | 0 (Strongly disagree) to 10 (Strongly agree) |
|  | Self-rated mental health | "How would you rate your overall mental health?" | 0 (Poor mental health) to 10 (Excellent mental health) |
| **Negative Well-Being** | |  |  |
|  | Feeling depressed | "Feeling down, depressed or hopeless" | 1 (Nearly every day) to 4 (Not at all) *(reverse-coded)* |
|  | Having little interest | "Little interest or pleasure in doing things" | Same as above |
|  | Feeling anxious | "Feeling nervous, anxious or on edge" | Same as above |
|  | Unable to control worry | "Not being able to stop or control worrying" | Same as above |
|  | Suffering | "To what extent are you suffering?" | 1 (A lot) to 4 (Not at all) *(reverse-coded)* |
| **Personality Traits** | | |  |
|  | Extraversion, Openness, Agreeableness, Conscientiousness, Neuroticism | *Assessed via the Ten-Item Personality Inventory (Gosling et al., 2003).* | 1 (Disagree strongly) to 7 (Agree strongly) |
| **Social Relationship Quality** | | |  |
|  | Relationship satisfaction | "My relationships are as satisfying as I would want them to be." | 0 (Strongly disagree) to 10 (Strongly agree) |
|  | Friendship contentment | "I am content with my friendships and relationships." | Same as above |
|  | Social support | "If you were in trouble, how often could you count on people in your life, like relatives or friends, to help you whenever you need them?" | 0 (Never) to 10 (Always) |
|  | Having people to show love | "How often do you show someone in your life that you love or care for them?" | Same as above |
|  | Loneliness | "How often do you feel lonely?" | 0 (Always) to 10 (Never) *(reverse-coded)* |

**Table S2.** Predicting Swedish Well-Being from demographics, social relationships, personality, and 10 SWB predictors with machine learning in cross-validated training (first row per model) and holdout (second row per model).

| Predictors | | *N*^predictors^ | *N^particpants^* | *Pearson r* | R^2^ | MAE | *RMSE* |
| --- | --- | --- | --- | --- | --- | --- | --- |
|  | Demographics | 55 | 11,098  2,768 | .45  .46 | .20  .21 | .68  .69 | .89  .91 |
|  | Personality traits | 5 | 11,843  2,961 | .62  .62 | .39  .38 | .60  .62 | .78  .81 |
|  | Social relationships quality | 5 | 11,879  2,968 | .70  .71 | .48  .50 | .54  .54 | .71  .73 |
|  | Demographics + personality traits | 60 | 10,949  2,724 | .68  .67 | .46  .44 | .55  .57 | .73  .76 |
|  | Demographics + social relationship quality | 60 | 10,976  2,731 | .73  .74 | .53  .55 | .51  .51 | .68  .68 |
|  | Personality traits + social relationship quality | 10 | 11,647  2,918 | .78  .78 | .61  .61 | .47  .48 | .62  .64 |
|  | Demographics + personality traits + social relationships quality | 65 | 10,779  2,688 | .79  .79 | .63  .63 | .45  .46 | .60  .62 |
|  | Demographics + personality traits + social relationships quality + 10 SWB predictors | 75 | 10,604  2,613 | .85  .86 | .73  .73 | .39  .49 | .52  .53 |

*Note:* First row per model = Cross-validated training. Second row per model = holdout.

Demographics include age, gender, region, political party orientation, income, employment status, number of adults in the household, marital status, urban vs rural living, born in Sweden/abroad, and religious service attendance. Personality traits include the big five traits. Social relationship quality includes friendship contentment, relationship satisfaction, loneliness, having people to help in trouble and having people to show love to. SWB predictors included variables that had above .30 correlation with the composite SWB, which were hope for the future, expecting good things to happen, self-rated physical health, belongingness in the country, gratitude, freedom, being able to afford expenses, difficulties living on present income, health when growing up and feeling incapable of doing things in life.

**Figure S14.** Beta (ꞵ) coefficients beyond 0 in the Lasso regression predicting Well-Being at *r* = .85 with penalty 0.01, including all variables with correlations to SWB above *r* = .30.
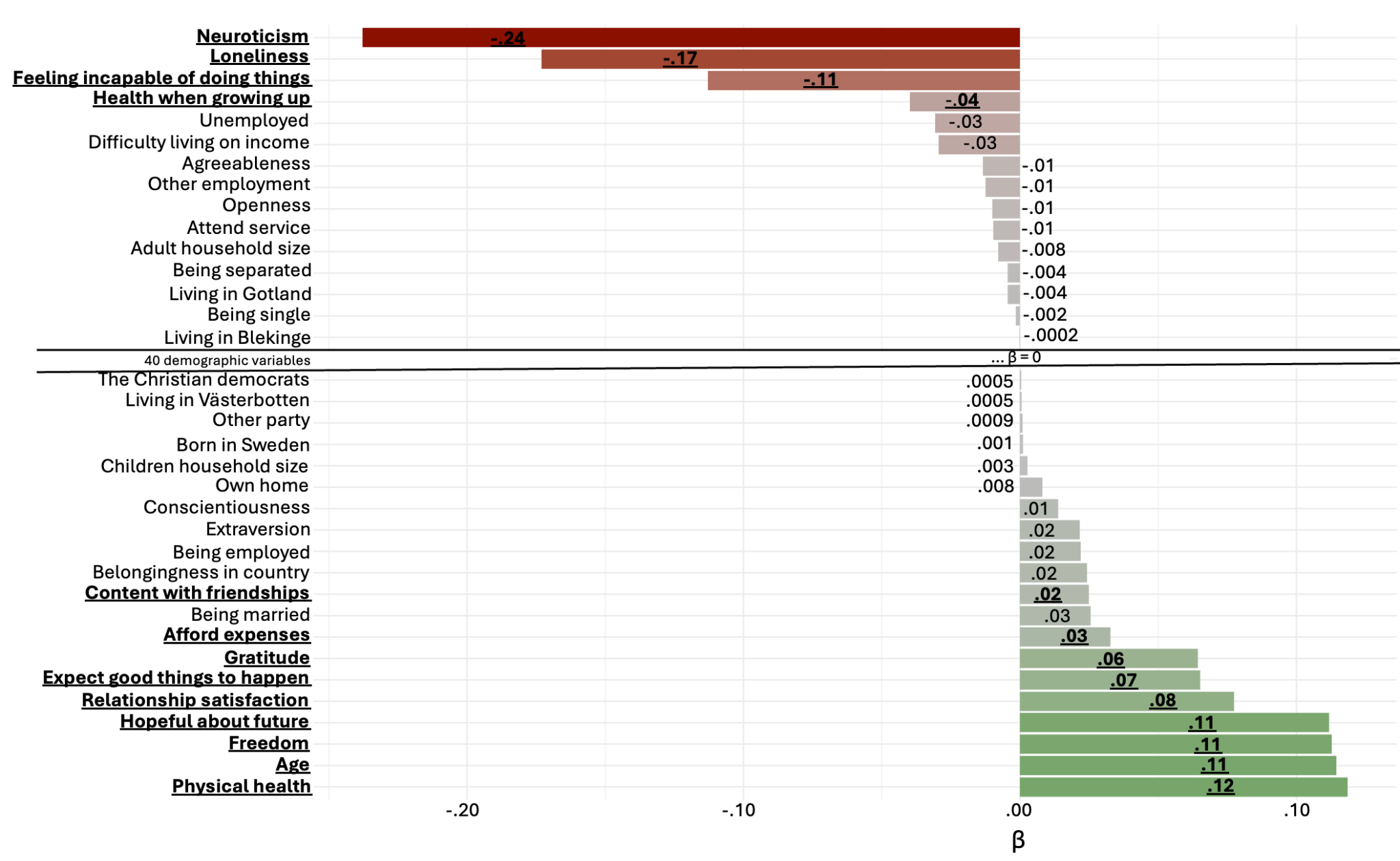


*Note:* The coefficients are ꞵ coefficients from the Lasso regression in the last row of Table S2 that used standardised predictors and criteria. Lasso regressions push non-necessary variables to 0 and keep meaningful (yet small) predictors above 0. The variables were ordered based on the strength of the beta coefficients. The penalty in the final model was 0.01. Bold predictors are those that remained non-zero when increasing the penalty to 0.1.

**Figure S15.** Beta (ꞵ) coefficients beyond 0 in the Lasso regression predicting Well-Being at *r* = .45 with penalty 0.01, using demographic predictors.


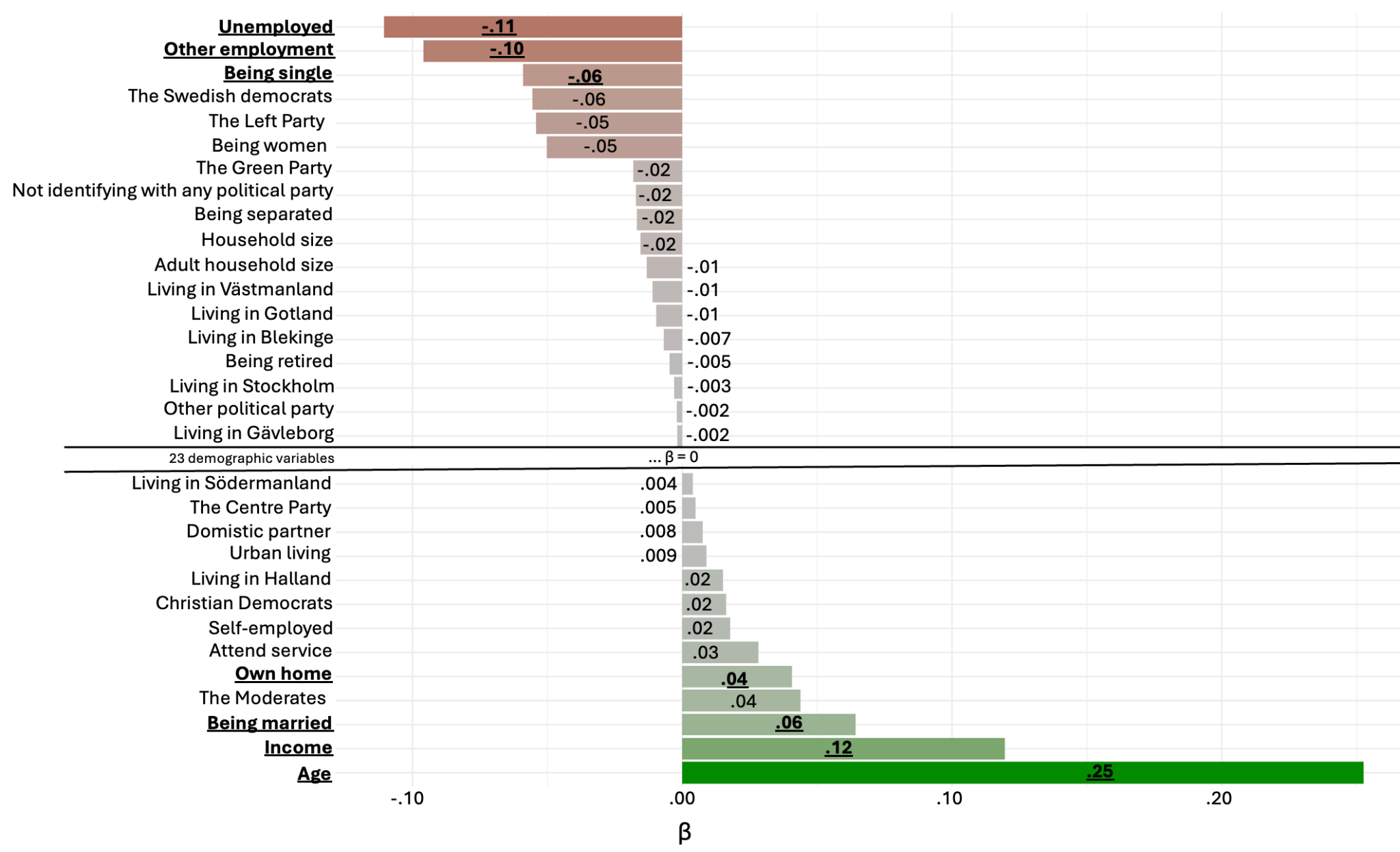


*Note:* The coefficients are ꞵ coefficients from the Lasso regression in the first row of Table 1 (i.e., demographics) that used standardised predictors and criteria. Lasso regressions push non-necessary variables to 0 and keep meaningful (yet small) predictors above 0. The variables were ordered based on the strength of the beta coefficients. The penalty in the final model was 0.01. Bold predictors are those that remained non-zero when increasing the penalty to 0.1.

**Figure S16.** Beta (ꞵ) coefficients beyond 0 in the Lasso regression predicting Well-Being at *r* = .62 with penalty 0.01, using personality predictors.
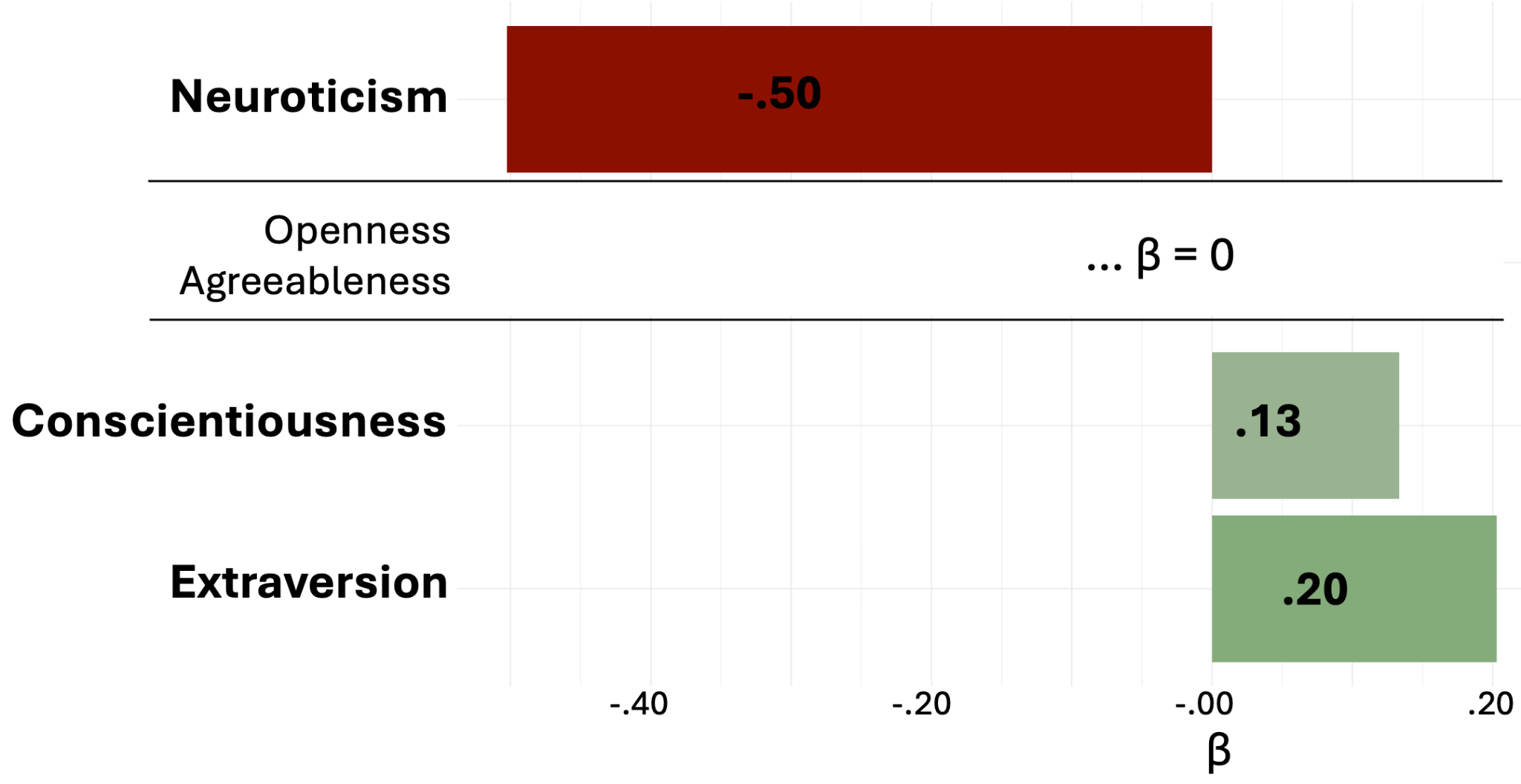


*Note:* The coefficients are ꞵ coefficients from the Lasso regression in the second row of Table 1 (i.e., personality traits) that used standardised predictors and criteria. Lasso regressions push non-necessary variables to 0 and keep meaningful (yet small) predictors above 0. The variables were ordered based on the strength of the beta coefficients. The penalty in the final model was 0.01. Bold predictors are those that remained non-zero when increasing the penalty to 0.1.

**Figure S17.** Beta (ꞵ) coefficients beyond 0 in the asso regression predicting Well-Being at *r* = .70 with penalty 0.001, using social relationship predictors.
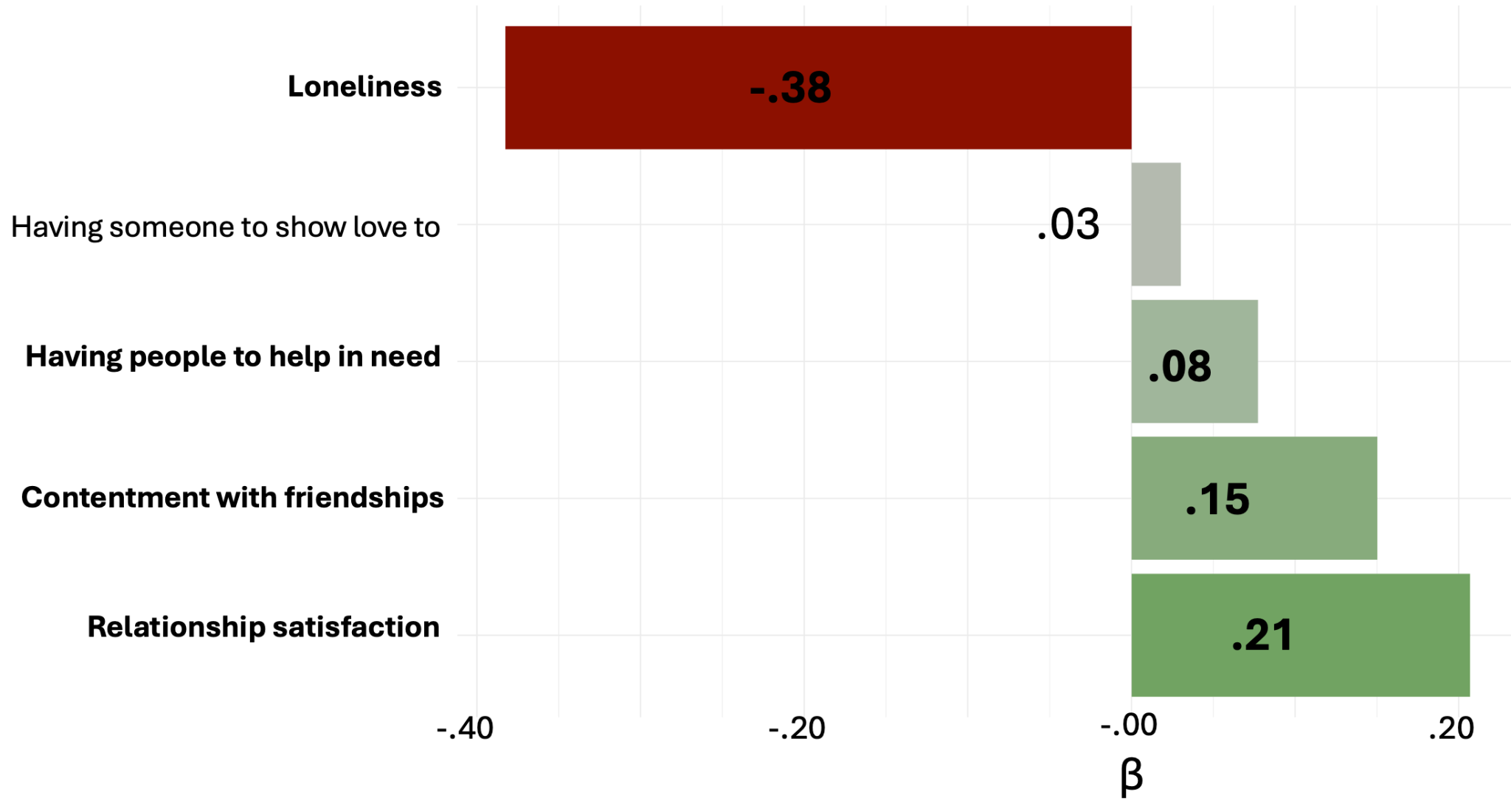


*Note:* The coefficients are ꞵ coefficients from the Lasso regression in the third row of Table 1 (i.e., social relationships quality) that used standardised predictors and criteria. Lasso regressions push non-necessary variables to 0 and keep meaningful (yet small) predictors above 0. The variables were ordered based on the strength of the beta coefficients. The penalty in the final model was 0.001. All predictors remained non-zero when increasing the penalty to 0.01.

**Figure S18.** Life evaluation trends in Sweden for different age groups between 2006 and 2023


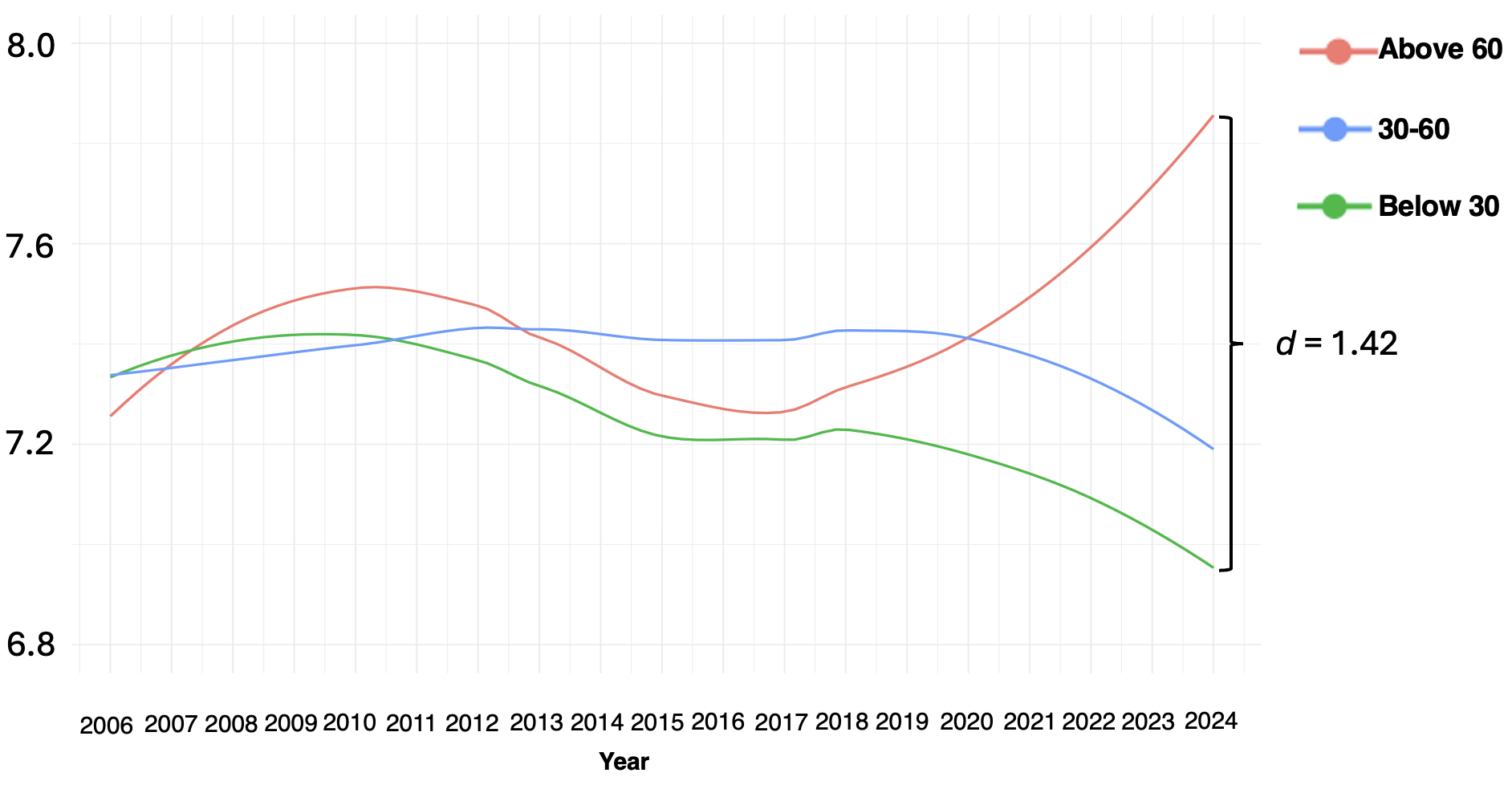


*Note*: The lines represent smoothed trends in average life evaluation scores across years for each age group. These trends were estimated using *locally estimated scatterplot smoothing* (LOESS). Data points are based on weighted means from the Gallup World Poll data. Cohen’s *d* was performed across the <30 and >60 age group in 2024.

**Figure S19.** Swedish political landscape based on factor analysis of 600+ expert ratings.


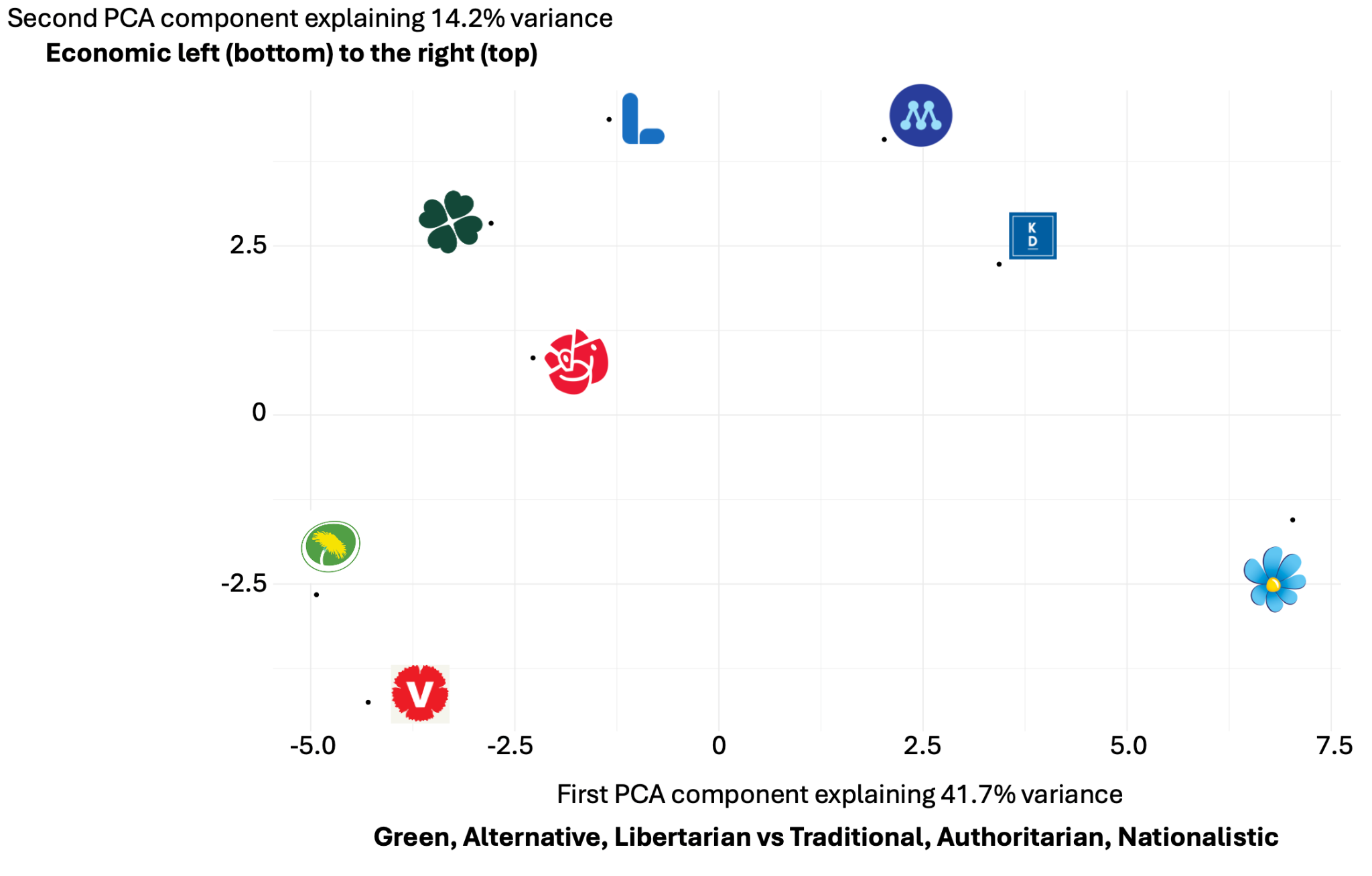
*Note***.** Swedish political landscape based on factor analysis of 600+ expert ratings. Each party is positioned based on its factor scores on the first two principal components derived from expert ratings in the 2024 Chapel Hill Expert Survey. The x-axis represents a GAL-TAN dimension (explaining 41.7% of the variance), and the y-axis reflects economic ideology from left (bottom) to right (top), accounting for an additional 14.2% of variance. Party logos are plotted at their respective positions.

**Methods.** Principal Component Analysis (PCA) was conducted on 45 political ideology and policy-related variables from the 2024 Chapel Hill Expert Survey (CHES), restricted to parties with complete data. The analysis then included 215 parties from 24 different countries. Variables included measures of general left–right economic orientation, cultural values (GAL-TAN), immigration, environmental attitudes, redistribution, deregulation, and others. All variables were standardized prior to analysis. The first two principal components were extracted and interpreted based on their post hoc Pearson correlations between each original variable and the principal component scores. The first component was strongly correlated with measures of GALTAN (*r* = .95), immigration policy (*r* = .95), women’s rights (*r* = .94), civil law order (*r* = .94) nationalism (*r* = .93), LBTQ rights (*r* = .93) and multiculturalism (*r* = .93). This component was interpreted as a GAL-TAN factor. The second component correlated most strongly with economic attitudes such as economic left to right (*r* = .77), deregulation (*r* = .76), and redistribution (*r* = .75). These components were used to plot the position of each Swedish political party in two-dimensional space in Figure S19.

**References**

Gosling, S. D., Rentfrow, P. J., & Swann Jr, W. B. (2003). A very brief measure of the Big-Five

personality domains. *Journal of Research in personality, 37*(6), 504-528.
